# Scalability of Asynchronous Networks Is Limited by One-to-One Mapping between Effective Connectivity and Correlations

**DOI:** 10.1371/journal.pcbi.1004490

**Published:** 2015-09-01

**Authors:** Sacha Jennifer van Albada, Moritz Helias, Markus Diesmann

**Affiliations:** 1 Institute of Neuroscience and Medicine (INM-6) and Institute for Advanced Simulation (IAS-6) and JARA BRAIN Institute I, Jülich Research Centre, Jülich, Germany; 2 Department of Psychiatry, Psychotherapy and Psychosomatics, Medical Faculty, RWTH Aachen University, Aachen, Germany; 3 Department of Physics, Faculty 1, RWTH Aachen University, Aachen, Germany; University College London, UNITED KINGDOM

## Abstract

Network models are routinely downscaled compared to nature in terms of numbers of nodes or edges because of a lack of computational resources, often without explicit mention of the limitations this entails. While reliable methods have long existed to adjust parameters such that the first-order statistics of network dynamics are conserved, here we show that limitations already arise if also second-order statistics are to be maintained. The temporal structure of pairwise averaged correlations in the activity of recurrent networks is determined by the effective population-level connectivity. We first show that in general the converse is also true and explicitly mention degenerate cases when this one-to-one relationship does not hold. The one-to-one correspondence between effective connectivity and the temporal structure of pairwise averaged correlations implies that network scalings should preserve the effective connectivity if pairwise averaged correlations are to be held constant. Changes in effective connectivity can even push a network from a linearly stable to an unstable, oscillatory regime and vice versa. On this basis, we derive conditions for the preservation of both mean population-averaged activities and pairwise averaged correlations under a change in numbers of neurons or synapses in the asynchronous regime typical of cortical networks. We find that mean activities and correlation structure can be maintained by an appropriate scaling of the synaptic weights, but only over a range of numbers of synapses that is limited by the variance of external inputs to the network. Our results therefore show that the reducibility of asynchronous networks is fundamentally limited.

## Author Summary

Neural networks have two basic components: their structural elements (neurons and synapses), and the dynamics of these constituents. The so-called *effective connectivity* combines both components to yield a measure of the actual influence of physical connections. Previous work showed effective connectivity to determine *correlations*, which quantify the co-activation of different neurons. Conversely, methods for estimating network structure from correlations have been developed. We here extend the range of networks for which the mapping between effective connectivity and correlations can be shown to be one-to-one, and clarify the conditions under which this equivalence holds. These findings apply to a class of networks that is often used, with some variations, to model the activity of cerebral cortex. Since the numbers of neurons and synapses in real mammalian brains are vast, such models tend to be reduced in size for simulation purposes. However, our findings imply that if we wish to retain the original dynamics including correlations, effective connectivity needs to be unchanged, from which we derive scaling laws for synaptic strengths and external inputs, and fundamental limits on the reducibility of network size. The work points to the importance of considering networks with realistic numbers of neurons and synapses.

## Introduction

While many aspects of brain dynamics and function remain unexplored, the numbers of neurons and synapses in a given volume are well known, and as such constitute basic parameters that should be taken seriously. Despite rapid advances in neural network simulation technology and increased availability of computing resources [1], memory and time constraints still lead to neuronal networks being routinely downscaled both on traditional architectures [2] and in systems dedicated to neural network simulation [3]. As synapses outnumber neurons by a factor of 10^3^ − 10^5^, these constitute the main constraint on network size. Computational capacity ranges from a few tens of millions of synapses on laptop or desktop computers, or on dedicated hardware when fully exploited [4, 5], to 10^12^ − 10^13^ synapses on supercomputers [6]. This upper limit is still about two orders of magnitude below the full human brain, underlining the need for downscaling in computational modeling. In fact, any brain model that approximates a fraction of the recurrent connections as external inputs is in some sense downscaled: the missing interactions need to be absorbed into the network and input parameters in order to obtain the appropriate statistics. Unfortunately, the implications of such scaling are usually not investigated.

**Fig 1:**
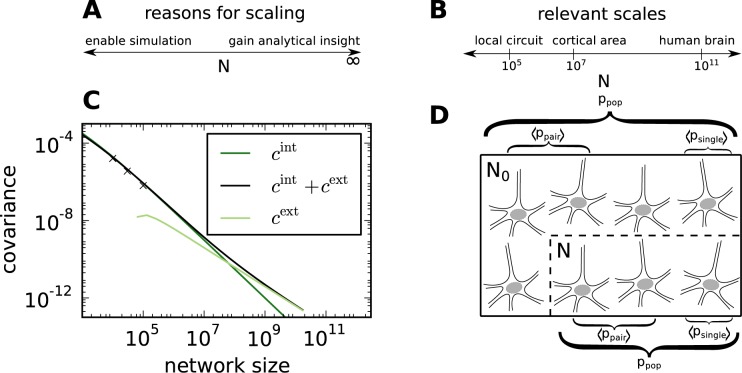
Framework for neural network scaling. **A** Downscaling facilitates simulations, while taking the *N* → ∞ limit often affords analytical insight. **B** Relevant scales. The local cortical microcircuit containing roughly 10^5^ neurons is the smallest network where the majority of the synapses (∼ 10^4^ per neuron) can be represented using realistic connection probabilities (∼ 0.1). **C** Results for the *N* → ∞ limit may not apply even for large networks. In this example, analytically determined intrinsic and extrinsic contributions to correlations between excitatory neurons are shown. The extrinsic contribution to the correlation between two neurons arises due common external input, and the intrinsic contribution due to fluctuations generated within the network (cf. [9] Eq 24). The intrinsic contribution falls off more rapidly than the extrinsic contribution, but nevertheless dominates up to large network sizes, here around 10^8^. The crosses indicate simulation results. Adapted from [9] Fig 7. **D** Scaling transformations may be designed to preserve average single-neuron or pairwise statistics for selected quantities, population statistics, or a combination of these. When average single-neuron and pairwise properties are preserved, the downscaled network of size *N* behaves to second order like a subsample of the full network of size *N*_0_.

The opposite type of scaling, taking the infinite size limit, is sometimes used in order to simplify equations describing the network (Fig 1A). Although this can lead to valuable insights, real networks in the human brain often contain on the order of 10^5^ − 10^7^ neurons (Fig 1B), too few to simplify certain equations in the limit of infinite size. This is illustrated in Fig 1C using as an example the intrinsic contribution to correlations due to fluctuations generated within the network, and the extrinsic contribution due to common external inputs to different neurons in random networks. Although the intrinsic contribution falls off more rapidly than the extrinsic one, it is the main contribution up to large network sizes (around 10^8^ for the given parameters). Therefore, taking the infinite size limit and neglecting the intrinsic contribution leads to the wrong conclusions: The small correlations in finite random networks cannot be explained by the network activity tracking the external drive [7], but rather require the consideration of negative feedback [8] that suppresses intrinsically generated and externally imprinted fluctuations alike [9].

Taking the infinite size limit for analytical tractability and downscaling to make networks accessible by direct simulation are two separate problems. We concentrate in the remainder of this study on such downscaling, which is often performed not only in neuroscience [10, 11, 12, 13] but also in other disciplines [14, 15, 16, 17]. Neurons and synapses may either be subsampled or aggregated [18]; here we focus on the former. One intuitive way of scaling is to ensure that the statistics of particular quantities of interest in the downscaled network match those of a subsample of the same size from the full network (Fig 1D). Alternatively, it may sometimes be useful to preserve the statistics of population sums of certain quantities, for instance population fluctuations.

We here focus on the preservation of mean population-averaged activities and pairwise averaged correlations in the activity. We consider both the size and temporal structure of correlations, but not distributions of mean activities and correlations across the network. Means and correlations present themselves as natural quantities to consider, because they are the first- and second-order and as such the most basic measures of the dynamics. If it is already difficult to preserve these measures, it is even less likely that preserving higher-order statistics will be possible, in view of their higher dimensionality. However, other choices are possible, for instance maintaining total input instead of output spike rates [19].

Besides being the most basic dynamical characteristics, means and correlations of neural activity are biologically relevant. Mean firing rates are important in many theories of network function [20, 21], and their relevance is supported by experimental results [22, 23]. For instance, neurons exhibit orientation tuning of spike rate in the visual system [24] and directional tuning in the motor system [25], and sustained rates are implicated in the working memory function of the prefrontal cortex [22]. Firing rates have also been shown to be central to pattern learning and retrieval in highly connected recurrent neural networks [21]. Furthermore, mean firing rates distinguish between states of arousal and attention [26, 27], and between healthy and disease conditions [28]. The relevance of correlations is similarly supported by a large number of findings. They are widely present; multi-unit recordings have revealed correlated neuronal activity in various animals and behavioral conditions [29, 30, 31]. Pairwise correlations were even shown to capture the bulk of the structure in the spiking activity of retinal and cultured cortical neurons [32]. They are also related to information processing and behavior. Synchronous spiking (corresponding to a narrow peak in the cross-correlogram) has for example been shown to occur in relation to behaviorally relevant events [33, 34, 35]. The relevance of correlations for information processing is further established by the fact that they can increase or decrease the signal-to-noise ratio of population signals [36, 37]. Moreover, correlations are important in networks with spike-timing-dependent plasticity, since they affect the average change in synaptic strengths [38]. Correspondingly, for larger correlations, stronger depression is needed for an equilibrium state with asynchronous firing and a unimodal weight distribution to exist in balanced random networks [39]. The level of correlations in neuronal activity has furthermore been shown to affect the spatial range of local field potentials (LFPs) effectively sampled by extracellular electrodes [40]. More generally, mesoscopic and macroscopic measures like the LFP and fMRI depend on interneuronal correlations [41]. Considering the wide range of dynamical and information processing properties affected by mean activities and correlations, it is important that they are accurately modeled.

We allow the number of neurons *N* and the number of incoming synapses per neuron *K* (the in-degree) to be varied independently, generalizing the common type of scaling where the connection probability is held constant so that *N* and *K* change proportionally. It is well known that reducing the number of neurons in asynchronous networks increases correlation sizes in inverse proportion to the network size [19, 42, 43, 44, 45]. However, the influence of the number of synapses on the correlations, including their temporal structure, is less studied. When reducing the number of synapses, one may attempt to recover aspects of the network dynamics by adjusting parameters such as the synaptic weights *J*, the external drive, or neurotransmitter release probabilities [11, 19]. In the present work, spike transmission is treated as perfectly reliable. We only adjust the synaptic weights and a combination of the neuronal threshold and the mean and variance of the external drive to make up for changes in *N* and *K*.

**Fig 2:**
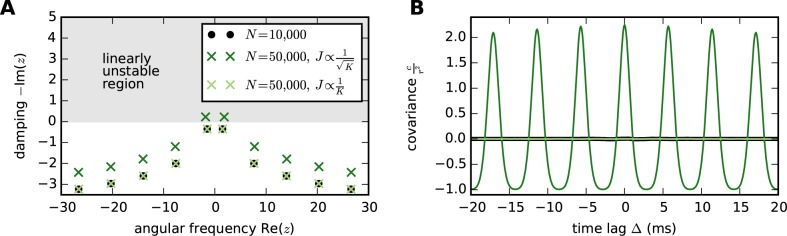
Transforming synaptic strengths *J* with the square root of the number of incoming synapses per neuron *K* (the in-degree) upon scaling of network size *N* changes correlation structure when mean and variance of the input current are maintained. A reference network of 10,000 inhibitory leaky integrate-and-fire neurons is scaled up to 50,000 neurons, fixing the connection probability and adjusting the external Poisson drive to keep the mean and variance of total (external plus internal) inputs fixed. Single-neuron parameters and connection probability are as in Table 2. Delays are 1 ms, mean and standard deviation of total inputs are 15 mV and 10 mV, respectively, and the reference network has *J* = 0.1 mV. Each network is simulated for 50 s. **A** Onset of oscillations induced by scaling of network size *N*, visualized by changes in the poles *z* of the covariance function in the frequency domain. Re(*z*) determines the frequency of oscillations and Im(*z*) their damping, such that -Im(*z*) > 0 means that small deviations from the fixed-point activity of the network grow with time [cf. Eq (76)]. The transformation $J\propto \frac{1}{K}$ preserves the poles, while $J\propto \frac{1}{\sqrt{K}}$ induces a Hopf bifurcation so that the scaled network is outside the linearly stable regime. **B** Covariance in the network where coupling strength *J* is scaled with the in-degree *K* matches that in the reference network, whereas large oscillations appear in the network scaled with $\sqrt{K}$. Colors as in **A**.

A few suggestions have been made for adjusting synaptic weights to numbers of synapses. In the balanced random network model, the asynchronous irregular (AI) firing often observed in cortex is explained by a domination of inhibition which causes a mean membrane potential below spike threshold, and sufficiently large fluctuations that trigger spikes [46]. In order to achieve such an AI state for a large range of network sizes, one choice is to ensure that input fluctuations remain similar in size, and adjust the threshold or a DC drive to maintain the mean distance to threshold. As fluctuations are proportional to *J* ^2^ *K* for independent inputs, this suggests the scaling

$$\begin{array}{c} J\propto \frac{1}{\sqrt{K}} \end{array}$$ (1)

proposed in [46]. Since the mean input to a neuron is proportional to *J* *K*, Eq (1) leads, all else being equal, to an increase of the population feedback with $\sqrt{K}$, changing the correlation structure of the network, as illustrated in Fig 2 for a simple network of inhibitory leaky integrate-and-fire neurons (note that in this example we fix the connection probability). This suggests the alternative [42, 44, 45]

$$\begin{array}{c} J\propto \frac{1}{K}, \end{array}$$ (2)

where now the variance of the external drive needs to be adjusted to maintain the total input variance onto neurons in the network.

For a given network size *N* and mean activity level, the size and temporal structure of pairwise averaged correlations are determined by the so-called *effective connectivity*, which quantifies the linear dependence of the activity of each target population on the activity of each source population. The effective connectivity is proportional to synaptic strength and the number of synapses a target neuron establishes with the source population, and additionally depends on the activity of the target neurons. Effective connectivity has previously been defined as “the experiment and time-dependent, simplest possible circuit diagram that would replicate the observed timing relationships between the recorded neurons” [47]. In our analysis we consider the stationary state, but at different times the network may be in a different state exhibiting a different effective connectivity. The definition of [47] highlights the fact that identical neural timing relationships can in principle occur in different physical circuits and vice versa. However, with a given model of interactions or coupling, the activity may allow a unique effective connectivity to be derived [48]. We define effective connectivity in a forward manner with knowledge of the physical connectivity as well as the form of interactions. We show in this study that with this model of interactions, and with independent external inputs, the activity indeed determines a unique effective connectivity, so that the forward and reverse definitions coincide. This complements the groundbreaking general insight of [47].

We consider networks of binary model neurons and networks of leaky integrate-and-fire (LIF) neurons with current-based synapses to investigate how and to what extent changes in network parameters can be used to preserve mean population-averaged activities and pairwise averaged correlations under reductions in the numbers of neurons and synapses. The parameters allowed to vary are the synaptic weights, neuronal thresholds, and the mean and variance of the external drive. We apply and extend the theory of correlations in randomly connected binary and LIF networks in the asynchronous regime developed in [7, 8, 9, 42, 45, 49, 50, 51, 52, 53], which explains the smallness and structure of correlations experimentally observed during spontaneous activity in cortex [54, 55], and we compare analytical predictions of correlations with results from simulations. The results are organized as follows. In “**Correlations uniquely determine effective connectivity: a simple example**” we provide an intuitive example that illustrates why the effective connectivity uniquely determines correlation structure. In “**Correlations uniquely determine effective connectivity: the general case**” we show that this one-to-one relationship generalizes to networks of several populations apart from degenerate cases. In “**Correlation-preserving scaling**” we conclude that, in general, only scalings that preserve the effective connectivity, such as *J* ∝ 1/*K*, are able to preserve correlations. In “**Limit to in-degree scaling**” we identify the limits of the resulting scaling procedure, demonstrating the restricted scalability of asynchronous networks. “**Robustness of correlation-preserving scaling**” shows that the scaling *J* ∝ 1/*K* can preserve correlations, within the identified restrictive bounds, for different networks either adhering to or deviating from the assumptions of the analytical theory. “**Zero-lag correlations in binary network**” investigates how to maintain the instantaneous correlations in a binary network, while “**Symmetric two-population spiking network**” considers the degenerate case of a connectivity with special symmetries, in which correlations may be maintained under network scaling without preserving the effective connectivity. Preliminary results have been published in abstract form [56].

## Results

### Correlations uniquely determine effective connectivity: A simple example

In this section we give an intuitive one-dimensional example to show that effective connectivity determines the shapes of the average pairwise cross-covariances and vice versa. For the following, we first introduce a few basic quantities. Consider a binary or spiking network consisting of several excitatory and inhibitory populations with potentially source- and target-type-dependent connectivity. For the spiking networks, we assume leaky integrate-and-fire (LIF) dynamics with exponential synaptic currents. The dynamics of the binary and LIF networks are respectively introduced in “**Binary network dynamics**” and “**Spiking network dynamics**”. We assume irregular network activity, approximated as Poissonian for the spiking network, with population means *ν*_*α*_. For the binary network, *ν* = ⟨*n*⟩ is the expectation value of the binary variable. For the spiking network, we absorb the membrane time constant into *ν*, defining *ν* = *τ*_m_ *r* where *r* is the firing rate of the population. The external drive can consist of both a DC component *μ*_*α*,ext_ and fluctuations with variance $\sigma_{\alpha ,\text{ext}}^{2}$, provided either by Poisson spikes or by a Gaussian current. The working points of each population, characterized by mean *μ*_*α*_ and variance $\sigma_{\alpha }^{2}$ of the combined input from within and outside the network, are given by

$$\begin{array}{ccc} \mu_{\alpha } & = & \sum_{\beta }J_{\alpha \beta }K_{\alpha \beta }\nu_{\beta }+\mu_{\alpha ,\text{ext}} \end{array}$$ (3)

$$\begin{array}{ccc} \sigma_{\alpha }^{2} & = & \sum_{\beta }J_{\alpha \beta }^{2}K_{\alpha \beta }\phi_{\beta }+\sigma_{\alpha ,\text{ext}.}^{2} \end{array}$$ (4)

$$\begin{array}{ccc} \text{with} & & \\ \phi & \equiv & \left\{ \begin{array}{cc} (1-\langle n\rangle )\left\langle n\right\rangle & \text{for}\;\text{binary}\\ \nu & \text{for}\;\text{LIF} \end{array},\right. \end{array}$$ (5)

where *J*_*αβ*_ is the synaptic strength from population *β* to population *α*, and *K*_*αβ*_ is the number of synapses per target neuron (the in-degree) for the corresponding projection (we use ≡ in the sense of “is defined as”). We call $\sigma_{\alpha ,\text{ext}}^{2}$ “external variance” in the following, and the remainder “internal variance”. The mean population activities are determined by *μ*_*α*_ and *σ*_*α*_ according to Eqs (39) and (67). Expressions for correlations in binary and LIF networks are given respectively in “**First and second moments of activity in the binary network**” and “**First and second moments of activity in the spiking network**”.

As a one-dimensional example, consider a binary network with a single population and vanishing transmission delays. The effective connectivity **W** is just a scalar, and the population-averaged autocovariance **a** and cross-covariance **c** are functions of the time lag Δ. We define the population-averaged effective connectivity as

$$\begin{array}{c} W=w(J,\mu ,\sigma )K, \end{array}$$ (6)

where *w*(*J*, *μ*, *σ*) is an effective synaptic weight that depends on the mean *μ* [Eq (3)] and the variance *σ* ^2^ [Eq (4)] of the input. For LIF networks, *w* = ∂*r*_target_/∂*r*_source_ is defined via Eq (68) and can be obtained as the derivative of Eq (67). Note that we treat the effective influence of individual inputs as independent. A more accurate definition of the population-level effective connectivity, beyond the scope of this paper, could be obtained by also considering combinations of inputs in the sense of a Volterra series [57]. When the dependence of *w* on *J* is linearized, the effective connectivity can be written as

$$\begin{array}{c} W=S(\mu ,\sigma )JK, \end{array}$$ (7)

where the susceptibility *S*(*μ*, *σ*) measures to linear order the effect of a unit input to a neuron on its outgoing activity. In our one-dimensional example, *W* quantifies the self-influence of an activity fluctuation back onto the population. Expressed in these measures, the differential equation for the covariance function [Eq (52)] takes the form

$$\begin{array}{c} \frac{\tau }{1-W}\frac{d}{d\Delta }c\left(\Delta \right)=-c\left(\Delta \right)+\frac{W}{1-W}\frac{a(\Delta )}{N}, \end{array}$$ (8)

with initial condition [from Eq (41)]

$$\begin{array}{c} (1-W)c\left(0\right)=\frac{Wa}{N}, \end{array}$$ (9)

which is solved by

$$\begin{array}{c} c\left(\Delta \right)=\frac{a}{N(1-W)}e^{\frac{W-1}{\tau }\Delta }-\frac{a}{N}e^{-\frac{\Delta }{\tau }}. \end{array}$$ (10)

Eq (10) shows that the effective connectivity *W* together with the time constant *τ* of the neuron (which we assume fixed under scaling) determines the temporal structure of the correlations. Furthermore, since a sum of exponentials cannot equal a sum of exponentials with a different set of exponents, the temporal structure of the correlations uniquely determines *W*. Hence we see that there is a one-to-one correspondence between *W* and the correlation structure if the time constant *τ* is fixed, which implies that preserving correlation structure under a reduction in the in-degrees *K* requires adjusting the effective synaptic weights *w*(*J*, *μ*, *σ*) such that the effective connectivity *W* is maintained. If, in addition, the mean activity ⟨*n*⟩ is kept constant this also fixes the variance *a* = ⟨*n*⟩(1 − ⟨*n*⟩). Eq (10) shows that, under these circumstances with *W* and *a* fixed, correlation sizes are determined by *N*.

### Correlations uniquely determine effective connectivity: The general case

More generally, networks consist of several neural populations each with different dynamic properties and with population-dependent transmission delays dαβ. Since this setting does not introduce additional symmetries, intuitively the one-to-one relationship between the effective connectivity and the correlations should still hold. We here show that, under certain conditions, this is indeed the case.

Instead of considering the covariance matrix in the time domain, for population-dependent dynamic properties we find it convenient to stay in the frequency domain. The influence of a fluctuating input on the output of the neuron can to lowest order be described by the transfer function *H*(*ω*). This quantity measures the amplitude and phase of the modulation of the neuronal activity given that the neuron receives a small sinusoidal perturbation of frequency *ω* in its input. The transfer function depends on the mean *μ* [Eq (3)] and the variance *σ* ^2^ [Eq (4)] of the input to the neuron. We here first consider LIF networks; in the Supporting Information we show how the results carry over to the binary model.

In “**First and second moments of activity in the spiking network**”, we give the covariance matrix including the autocovariances in the frequency domain, $\bar{\text{C}}(\omega )=\text{C}(\omega )+\text{A}(\omega )$, as

$$\begin{array}{c} \bar{C}\left(\omega \right)={\left(𝟙-M(\omega )\right)}^{-1}A{\left(𝟙-M^{T}\left(-\omega \right)\right)}^{-1}, \end{array}$$ (11)

where **M** has elements *H*_*αβ*_(*ω*)*W*_*αβ*_. If $\bar{\text{C}}(\omega )$ is invertible, we can expand the inverse of Eq (11) to obtain

$$\begin{array}{ccc} {\bar{C}}_{\alpha \beta }^{-1}\left(\omega \right) & = & \sum_{\gamma }({𝟙}_{\alpha \gamma }-M_{\gamma \alpha }\left(-\omega \right))A_{\gamma }^{-1}({𝟙}_{\gamma \beta }-M_{\gamma \beta }\left(\omega \right))\\ & = & \delta_{\alpha \beta }\;(1-W_{\alpha \alpha }\frac{e^{i\omega d_{\alpha \alpha }}}{1-i\omega \tau_{\alpha }})A_{\alpha }^{-1}(1-W_{\alpha \alpha }\frac{e^{-i\omega d_{\alpha \alpha }}}{1+i\omega \tau_{\alpha }})\\ & + & \left(\delta_{\alpha \beta }-1\right)[(1-W_{\alpha \alpha }\frac{e^{i\omega d_{\alpha \alpha }}}{1-i\omega \tau_{\alpha }})A_{\alpha }^{-1}W_{\alpha \beta }\frac{e^{-i\omega d_{\alpha \beta }}}{1+i\omega \tau_{\alpha }}\\ & + & W_{\beta \alpha }\frac{e^{i\omega d_{\beta \alpha }}}{1-i\omega \tau_{\beta }}A_{\beta }^{-1}(1-W_{\beta \beta }\frac{e^{-i\omega d_{\beta \beta }}}{1+i\omega \tau_{\beta }})]\\ & + & \sum_{\gamma \neq \alpha ,\beta }W_{\gamma \alpha }\frac{e^{i\omega d_{\gamma \alpha }}}{1-i\omega \tau_{\gamma }}A_{\gamma }^{-1}W_{\gamma \beta }\frac{e^{-i\omega d_{\gamma \beta }}}{1+i\omega \tau_{\gamma }}, \end{array}$$ (12)

where we assumed the transfer function to have the form $H(\omega )=\frac{e^{-i\omega d_{\alpha \beta }}}{1+i\omega \tau_{\alpha }},$ which is often a good approximation for the LIF model [45]. In the second step we distinguish terms that only contribute on the diagonal (*α* = *β*), those that only contribute off the diagonal (*α* ≠ *β*), and those that contribute in either case. For *α* = *β*, only the first and last terms contribute, and we get

$$\begin{array}{ccc} {\bar{C}}_{\alpha \alpha }^{-1} & = & A_{\alpha }^{-1}\\ & - & \frac{W_{\alpha \alpha }}{A_{\alpha }}(\frac{e^{-i\omega d_{\alpha \alpha }}}{1+i\omega \tau_{\alpha }}+\frac{e^{i\omega d_{\alpha \alpha }}}{1-i\omega \tau_{\alpha }})\\ & + & \sum_{\gamma }\frac{W_{\gamma \alpha }^{2}A_{\gamma }^{-1}}{1+\omega^{2}\tau_{\gamma }^{2}}. \end{array}$$ (13)

If we want to preserve $\bar{\text{C}}$, this fixes *A*_*α*_ and thereby also *W*_*αα*_, since it multiplies terms with unique *ω*-dependence. For *α* ≠ *β*, we obtain

$$\begin{array}{ccc} {\bar{C}}_{\alpha \beta }^{-1} & = & \frac{W_{\alpha \beta }}{A_{\alpha }}e^{-i\omega d_{\alpha \beta }}(-\frac{1}{1+i\omega \tau_{\alpha }}+W_{\alpha \alpha }\frac{e^{i\omega d_{\alpha \alpha }}}{1+\omega^{2}\tau_{\alpha }^{2}})\\ & + & \frac{W_{\beta \alpha }}{A_{\beta }}e^{i\omega d_{\beta \alpha }}(-\frac{1}{1-i\omega \tau_{\beta }}+W_{\beta \beta }\frac{e^{-i\omega d_{\beta \beta }}}{1+\omega^{2}\tau_{\beta }^{2}})\\ & + & \sum_{\gamma \neq \alpha ,\beta }\frac{W_{\gamma \alpha }W_{\gamma \beta }}{A_{\gamma }}\frac{e^{i\omega (d_{\gamma \alpha }-d_{\gamma \beta })}}{1+\omega^{2}\tau_{\gamma }^{2}}. \end{array}$$ (14)

With *A*_*α*_ fixed, this additionally fixes *W*_*αβ*_, in view of the unique *ω*-dependence it multiplies.

Since $\text{C}(\omega )=\bar{\text{C}}(\omega )-\text{A}$, a constraint on **A** necessary for preserving $\bar{\text{C}}(\omega )$ may not translate into the same constraint when we only require the cross-covariances **C**(*ω*) to be preserved. However, **C**(*ω*) and $\bar{\text{C}}(\omega )$ have identical *ω*-dependence, as they differ only by constants on the diagonal (approximating autocorrelations as delta functions in the time domain [45]). To derive conditions for preserving **C**(*ω*), we therefore ignore the constraint on **A** but still require the *ω*-dependence to be unchanged. A potential transformation leaving the *ω*-dependent terms in both Eqs (13) and (14) unchanged is *A*_*α*_ → *kA*_*α*_, *W*_*αβ*_ → *kW*_*αβ*_, *W*_*αα*_ → *kW*_*αα*_, but this only works if *τ*_*α*_ = *τ*_*γ*_, *d*_*αα*_ − *d*_*αβ*_ = *d*_*γα*_ − *d*_*γβ*_ for some *γ*, and if the terms for the corresponding *γ* are also transformed to offset the change in $W_{\alpha \alpha }W_{\alpha \beta }A_{\alpha }^{-1}$; or if some of the entries of **W** vanish. The *ω*-dependence of $\bar{\text{C}}$ and **C** would otherwise change, showing that, at least in the absence of such symmetries in the delays or time constants, or zeros in the effective connectivity matrix (i.e., absent connections at the population level, or inactive populations), there is a one-to-one relationship between covariances and effective connectivity. Hence, preserving the covariances requires preserving **A** and **W** except in degenerate cases. Note that the autocovariances and hence the firing rates can be changed while affecting only the size but not the shape of the correlations, but that the correlation shapes determine **W**.

Even in case of identical transfer functions across populations, including in particular equal transmission delays and identical *τ*, the one-to-one correspondence between effective connectivity and correlations can be demonstrated except for a narrower set of degenerate cases. The argument for *d* = 0 proceeds in the time domain along the same lines as “**Correlations uniquely determine effective connectivity: a simple example**”, using the fact that for a population-independent transfer function, the correlations can be expressed in terms of the eigenvalues and eigenvectors of the effective connectivity matrix (cf. “**First and second moments of activity in the binary network**” and “**First and second moments of activity in the spiking network**”). For general delays, a derivation in the frequency domain can be used. Through these arguments, we show in the Supporting Information that the one-to-one correspondence holds at least if **W** is diagonalizable and has no eigenvalues that are zero or degenerate.

### Correlation-preserving scaling

If the working point (*μ*, *σ*) is maintained, the one-to-one correspondence between the effective connectivity and the correlations implies that requiring unchanged average covariances leaves no freedom for network scaling except for a possible trade-off between in-degrees and synaptic weights. In the linear approximation *W*(*J*, *μ*, *σ*) = *S*(*μ*, *σ*)*JK*, this trade-off is *J* ∝ 1/*K*.

**Fig 3:**
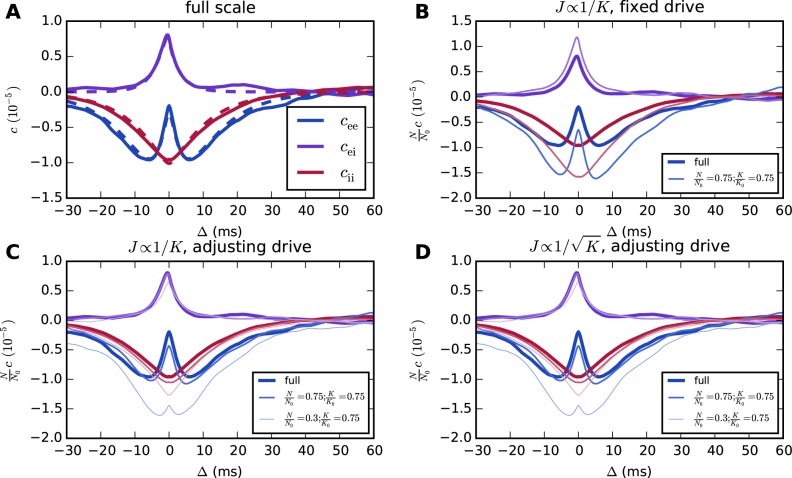
Correlations from theory and simulations for a two-population binary network with asymmetric connectivity. **A** Average pairwise cross-covariances from simulations (solid curves) and Eq (55) (dashed curves). **B** Naive scaling with *J* ∝ 1/*K* but without adjustment of the external drive changes the correlation structure. **C** With an appropriate adjustment of the external drive (*σ*_ex_ = 53.4, *σ*_ix_ = 17.7), scaling synaptic weights as *J* ∝ 1/*K* is able to preserve correlation structure as long as *N* and *K* are reduced by comparable factors. **D** The same holds for $J\propto 1/\sqrt{K}$ (*μ*_ex_ = 43.3, *μ*_ix_ = 34.6, *σ*_ex_ = 46.2, *σ*_ix_ = 15.3), but the susceptibility *S* is increased by about 20% already for *N* = 0.75 *N*_0_ in this case. In **B**, **C**, and **D**, results of simulations are shown. The curves in **C** and **D** are identical because internal inputs, the standard deviation of the external drive, and the distance to threshold due to the DC component of the drive in **D** are exactly $\sqrt{\frac{K}{K_{0}}}$ times those in **C**. Hence, identical realizations of the random numbers for the connectivity and the Gaussian external drive cause the total inputs to the neurons to exceed the threshold at exactly the same points in time in the two simulations. The simulated time is 30 s, and the population activity is sampled at a resolution of 0.3 ms.

When this scaling is implemented naively without adjusting the external drive to recover the original working point, the covariances change, as illustrated in Fig 3B for a two-population binary network with parameters given in Table 1. The results of *J* ∝ 1/*K* scaling with appropriate adjustment of the external drive are shown in Fig 3C. The scaling shown in Fig 3B also increases the mean activities (E: from 0.16 to 0.23, I: from 0.07 to 0.11), whereas that in Fig 3C preserves them.

**Table 1:** Parameters of the asymmetric binary network.

numbers of neurons	*N*_e_, *N*_i_	5000, 5000
neuron time constant	*τ*	10 ms
threshold	*θ*	0
connection probabilities	*p*_ee_, *p*_ei_, *p*_ie_, *p*_ii_	0.1, 0.2, 0.3, 0.4
transmission delay	*d*	0.1 ms
synaptic weights	*J*_ee_, *J*_ei_, *J*_ie_, *J*_ii_	3, −5, 3, −6
mean external drive	*m*_ex_, *m*_ix_	50, 40
SD of external drive	*σ*_ex_, *σ*_ix_	60, 50

If one relaxes the constraint on the working point while still requiring mean activities to be preserved, the network does have additional symmetries due to the fact that only some combination of *μ* and *σ* needs to be fixed, rather than each of these separately. This combination is more easily determined for binary than for LIF networks, for which the mean firing rates depend on *μ* and *σ* in a complex manner [cf. Eq (67)]. When the derivative of the gain function is narrow (e.g., having zero width in the case of the Heaviside function used here) compared to the input distribution, the mean activities of binary networks depend only on (*μ* − *θ*)/*σ* [9]. Changing *σ* while preserving (*μ* − *θ*)/*σ* leads for a Heaviside gain function to a new susceptibility *S*′ = (*σ*/*σ*′)*S* [cf. Eq (43)]. For constant *K*, if the standard deviation of the external drive is changed proportionally to the internal standard deviation, we have *σ* ∝ *J* and thus *J*′ *S*′ = *JS*, implying an insensitivity of the covariances to the synaptic weights *J* [52]. In particular, this symmetry applies in the absence of an external drive. When *K* is altered, this choice for adjusting the external drive causes the covariances to change. However, adjusting the external drive such that *σ*′/*σ* = (*J*′ *K*′)/(*JK*), the change in *S* is countered to preserve **W** and correlations. This is illustrated in Fig 3D for $J\propto 1/\sqrt{K}$, which is another natural choice, as it preserves the internal variance if one ignores the typically small contribution of the correlations to the input variance ([9] Fig 3D illustrates the smallness of this contribution for an example network). This is only one of a continuum of possible scalings preserving mean activities and covariances (within the bounds described in the following section) when the working point and hence the susceptibility are allowed to change.

### Limit to in-degree scaling

We now show that both the scaling *J* ∝ 1/*K* for LIF networks (for which we do not consider changes to the working point, as analytic expressions for countering these changes are intractable), and correlation-preserving scalings for binary networks (where we allow changes to the working point that preserve mean activities) are applicable only up to a limit that depends on the external variance.

For the binary network, assume a generic scaling *K*′ = *κK*, *J*′ = *ιJ* and a Heaviside gain function. We denote variances due to inputs from within the network and due to the external drive respectively by $\sigma_{\text{int}}^{2}$ and $\sigma_{\text{ext}}^{2}.$ The preservation of the mean activities implies *S*′ = (*σ*/*σ*′)*S* as above, where $\sigma^{2}=\sigma_{\text{ext}}^{2}+\sigma_{\text{int}}^{2}$. To keep *SJK* fixed we thus require

$$\begin{array}{ccc} \sigma_{\text{int}}^{2}{\;}^{\prime }+\sigma_{\text{ext}}^{2}{\;}^{\prime } & = & {\left(\iota \kappa \right)}^{2}(\sigma_{\text{int}}^{2}+\sigma_{\text{ext}}^{2})\\ \sigma_{\text{ext}}^{2}{\;}^{\prime } & = & \iota^{2}\kappa [(\kappa -1)\sigma_{\text{int}}^{2}+\kappa \sigma_{\text{ext}}^{2}], \end{array}$$ (15)

where we have used $\sigma_{\text{int}}^{\prime }\approx \iota \sqrt{\kappa }\sigma_{\text{int}}$ in the second line. For *σ*_ext_ = 0 this scaling only works for *κ* > 1, i.e., increasing instead of decreasing the in-degrees. More generally, the limit to downscaling occurs when $\sigma_{\text{ext}}^{\prime }=0,$ or

$$\begin{array}{c} \kappa =\frac{\sigma_{\text{int}}^{2}}{\sigma_{\text{int}}^{2}+\sigma_{\text{ext}}^{2}}, \end{array}$$ (16)

independent of the scaling of the synaptic weights. Thus, larger external and smaller internal variance before scaling allow a greater reduction in the number of synapses. The in-degrees of the example network of Fig 3 could be maximally reduced to 73%. Note that *ι* could in principle be chosen in a *κ*-dependent manner such that $\sigma_{\text{ext}}^{2}$ is fixed or increased instead of decreased upon downscaling, namely $\iota \geq \sqrt{\frac{\sigma_{\text{ext}}^{2}}{\kappa^{2}\sigma_{\text{ext}}^{2}+\kappa (\kappa -1)\sigma_{\text{int}}^{2}}}$. However, Eq (16) is still the limit beyond which this fails, as *ι* then diverges at that point.

Note that the limit to the in-degree scaling also implies a limit on the reduction in the number of neurons for which the scaling equations derived here allow the correlation structure to be preserved, as a greater reduction of *N* compared to *K* increases the number of common inputs neurons receive and thereby the deviation from the assumptions of the diffusion approximation. This is shown by the thin curves in Fig 3C,3D.

Now consider correlation-preserving scaling of LIF networks. Reduced *K* with constant *JK* does not affect mean inputs [cf. Eq (3)] but increases the internal variance according to Eq (4). To maintain the working point (*μ*, *σ*), it is therefore necessary to reduce the variance of the external drive. When the drive consists of excitatory Poisson input, one way of keeping the mean external drive constant while changing the variance is to add an inhibitory Poisson drive. With *K*′ = *K*/*ι* and *J*′ = *ιJ*, the change in internal variance is $(\iota -1)\sigma_{\text{int}}^{2}$, where $\sigma_{\text{int}}^{2}$ is the internal variance due to input currents in the full-scale model. This is canceled by an opposite change in $\sigma_{\text{ext}}^{2}$ by choosing excitatory and inhibitory Poisson rates

$$\begin{array}{c} r_{e,\text{ext}}=r_{e,0}+\frac{\left(1-\iota \right)\sigma_{\text{int}}^{2}}{\tau_{m}J_{\text{ext}}^{2}(1+g)}, \end{array}$$ (17)

$$\begin{array}{c} r_{i,\text{ext}}=\frac{\left(1-\iota \right)\sigma_{\text{int}}^{2}}{\tau_{m}J_{\text{ext}}^{2}g\left(1+g\right)}, \end{array}$$ (18)

where *r*_e,0_ is the Poisson rate in the full-scale model, and the excitatory and inhibitory synapses have weights *J*_ext_ and −*g* *J*_ext_, respectively. Eqs (17) and (18) match Eq (E.1) in [45] except for the 1 + *g* in the denominator, which was there erroneously given as 1+*g* ^2^. Since downscaling *K* implies *ι* > 1, it is seen that the required rate of the inhibitory inputs is negative. Therefore, this method only allows upscaling. An alternative is to use a balanced Poisson drive with weights *J*_ext_ and − *J*_ext_, choosing the rate of both excitatory and inhibitory inputs to generate the desired variance, and adding a DC drive *I*_ext_ to recover the mean input,

$$\begin{array}{ccc} r_{e,\text{ext}}=r_{i,\text{ext}} & = & \frac{r_{e,0}}{2}+\frac{\left(1-\iota \right)\sigma_{\text{int}}^{2}}{2\tau_{m}J_{\text{ext}}^{2}}, \end{array}$$ (19)

$$\begin{array}{ccc} I_{\text{ext}} & = & \tau_{m}r_{e,0}J_{\text{ext}}. \end{array}$$ (20)

In this manner, the network can be downscaled up to the point where the variance of the external drive vanishes. Substituting this condition into Eq (15), the same expression for the minimal in-degree scaling factor Eq (16) is obtained as for the binary network.

### Robustness of correlation-preserving scaling

**Fig 4:**
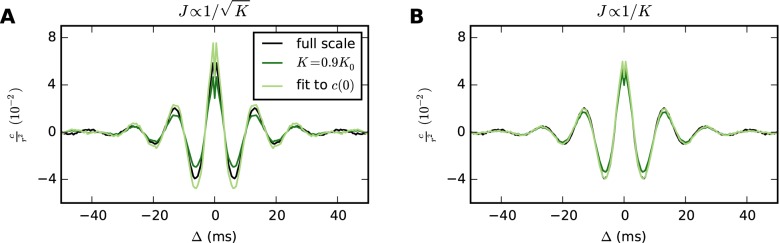
Within the restrictive bounds imposed by Eq (16), preserving effective connectivity can preserve correlations also in a complex network. Simulation results for the cortical microcircuit at full scale and with in-degrees reduced to 90%. Synaptic strengths are scaled as indicated, and the external drive is adjusted to restore the working point. Mean pairwise cross-covariances are shown for population 2/3E. Qualitatively identical results are obtained within and across other populations. The simulation duration is 30 s and covariances are determined with a resolution of 0.5 ms. To enable downscaling with *J* ∝ 1/*K*, the excitatory Poisson input of the original implementation of [58] is replaced by balanced inhibitory and excitatory Poisson input with a DC drive according to Eqs (19) and (20). **A** Scaling synaptic strengths as $J\propto 1/\sqrt{K}$ changes the mean covariance. Light green curve: stretching the covariance of the scaled network along the vertical axis to match the zero-lag correlation of the full-scale network shows that not only the size but also the temporal structure of the covariance is affected. **B** Scaling synaptic strengths as *J* ∝ 1/*K* closely preserves the covariance of the full-scale network. However, note that this scaling is only applicable down to the in-degree scaling factor given by Eq (16), which for this example is approximately 0.9.

In this section, we show that the scaling *J* ∝ 1/*K*, which maintains the population-level feedback quantified by the effective connectivity, can preserve correlations (within the bounds given in “**Limit to in-degree scaling**”) under fairly general conditions. To this end, we consider two types of networks: 1. a multi-layer cortical microcircuit model with distributed in- and out-degrees and lognormally distributed synaptic strengths (cf. “**Network structure and notation**”); 2. a two-population LIF network with different mean firing rates (parameters in Table 2). For both types of models, we contrast the scaling *J* ∝ 1/*K* with $J\propto 1/\sqrt{K}$, in each case maintaining the working point given by Eqs (3) and (4). Fig 4 illustrates that the former closely preserves average pairwise cross-covariances in the cortical microcircuit model, whereas the latter changes both their size and temporal structure.

**Table 2:** Full-scale parameters of the two-population spiking networks used to demonstrate the robustness of *J* ∝ 1/*K* scaling to mean firing rates. The two networks are distinguished by their external drives.

number of excitatory neurons	*N*	8000
relative inhibitory population size	*γ*	0.25
membrane time constant	*τ*_m_	20 ms
synaptic time constant	*τ*_s_	2 ms
refractory period	*τ*_ref_	2 ms
membrane resistance	*R*_m_	20 MΩ
resting and reset potential	*V*_r_	0 mV
threshold	*θ*	15 mV
connection probability	*p*	0.1
transmission delay	*d*	3 ms
excitatory synaptic weight	*J*	0.1 mV
relative inhibitory synaptic weight	*g*	5
mean and standard deviation of external drive	(*μ*_ext_, *σ*_ext_)	(10 mV, 5 mV);
		(25 mV, 20 mV)

**Fig 5:**
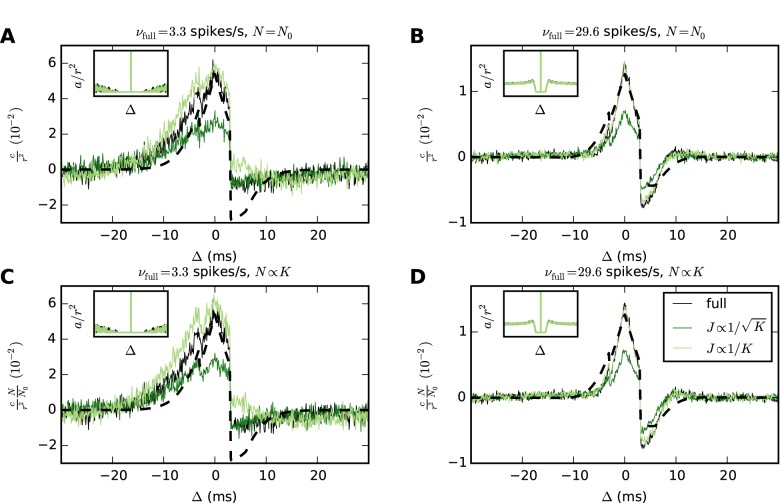
Scaling synaptic strengths as *J* ∝ 1/*K* can preserve correlations in networks with widely different firing rates. Results of simulations of a LIF network consisting of one excitatory and one inhibitory population (Table 2). Average cross-covariances are determined with a resolution of 0.1 ms and are shown for excitatory-inhibitory neuron pairs. Each network receives a balanced Poisson drive with excitatory and inhibitory rates both given by $\sigma_{\text{ext}}^{2}/(2\;\tau_{\text{m}}\;J^{2})$, where $\sigma_{\text{ext}}^{2}$ is chosen to maintain the working point of the full-scale network. The synaptic strengths for the external drive are 0.1 mV and −0.1 mV for excitatory and inhibitory synapses, respectively. A DC drive with strength *μ*_ext_ is similarly adjusted to maintain the full-scale working point. All networks are simulated for 100 s. For each population, cross-covariances are computed as averages over all neuron pairs across two disjoint groups of 𝓝 × 1000 neurons, where 𝓝 is the scaling factor for the number of neurons (a given pair has one neuron in each group). Autocovariances are computed as averages over 100 neurons in each population. **A**, **B** Reducing in-degrees *K* to 50% while the number of neurons *N* is held constant, *J* ∝ 1/*K* closely preserves both the size and the shape of the covariances, while $J\propto 1/\sqrt{K}$ diminishes their size. **C**, **D** Reducing both *N* and *K* to 50%, covariance sizes scale with 1/*N* for *J* ∝ 1/*K* but with a different factor for $J\propto 1/\sqrt{K}$. Dashed curves represent theoretical predictions. The insets show mean autocovariances for time lags Δ ∈ (−30, 30) ms.

Fig 5 demonstrates the robustness of *J* ∝ 1/*K* scaling to the firing rate of the network. In this example, both the full-scale network and the downscaled networks receive a balanced Poisson drive producing the desired variance, while the mean input is provided by a DC drive. By changing the parameters of the external drive, we create two networks each with irregular spiking but with widely different mean rates (3.3 spikes/s and 29.6 spikes/s). Downscaling only the number of synapses but not the number of neurons, both the temporal structure and the size of the correlations are closely preserved. Reducing the in-degrees and the number of neurons *N* by the same factor, the correlations are scaled by 1/*N*. Hence, the correlations of the full-scale network of size *N*_0_ can be estimated simply by multiplying those of the reduced network by *N*/*N*_0_. In contrast, $J\propto 1/\sqrt{K}$ changes correlation sizes even when *N* is held constant, and combined scaling of *N* and *K* can therefore not simply be compensated for by the factor *N*/*N*_0_. In the high-rate network, the spiking statistics of the neurons is non-Poissonian, as seen from the gap in the autocorrelations (insets in Fig 5B, 5D). Nevertheless, *J* ∝ 1/*K* preserves the correlations more closely than $J\propto 1/\sqrt{K}$, showing that the predicted scaling properties hold beyond the strict domain of validity of the underlying theory.

### Zero-lag correlations in binary network

Although it is not generally possible to keep mean activities and correlations invariant upon downscaling, transformations may be found when only one aspect of the correlations is important, such as their zero-lag values. We illustrate this using a simple, randomly connected binary network of *N* excitatory and *γN* inhibitory binary neurons, where each neuron receives *K* = *pN* excitatory and *γK* inhibitory inputs. The parameters are given in Table 3. The linearized effective connectivity matrix for this example is

$$\begin{array}{c} W=S\left(\mu ,\sigma \right)JK(\begin{array}{cc} 1 & -\gamma g\\ 1 & -\gamma g \end{array}). \end{array}$$ (21)

**Table 3:** Parameters of the symmetric binary network.

number of excitatory neurons	*N*	1000
relative inhibitory population size	*γ*	1
neuron time constant	*τ*	10 ms
threshold	*θ*	−3
connection probability	*p*	0.2
transmission delay	*d*	0.1 ms
excitatory synaptic weight	*J*	$1/\sqrt{1000}$
relative inhibitory synaptic weight	*g*	3
external drive	*m*_x_	0

When the threshold *θ* is ≤ 0, the network is spontaneously active without external inputs. In the diffusion approximation and assuming stationarity, the mean zero-lag cross-covariances between pairs of neurons from each population can be estimated from Eq (41) (see also [52])

$$\begin{array}{c} \left[(\begin{array}{cc} 1 & 0\\ 0 & 1 \end{array})-\frac{W_{e}}{2}(\begin{array}{cc} 2-\gamma g & -\gamma g\\ 1 & 1-2\gamma g \end{array})](\begin{array}{c} c_{\text{ee}}\\ c_{\text{ii}} \end{array})=\frac{W_{e}a}{N}(\begin{array}{c} 1\\ -g \end{array}\right) \end{array}$$

$$\begin{array}{c} c_{\text{ei}}=c_{\text{ie}}=\frac{1}{2}\left(c_{\text{ee}}+c_{\text{ii}}\right), \end{array}$$ (22)

where the subscripts e and i respectively denote excitatory and inhibitory populations. Moreover, *W*_e_ is the effective excitatory coupling,

$$\begin{array}{c} W_{e}=S\left(\mu ,\sigma \right)JK, \end{array}$$ (23)

with *S* the susceptibility as defined in Eq (43). Furthermore, *a* is the variance of the single-neuron activity,

$$\begin{array}{c} a=\langle n\rangle \left(1-\langle n\rangle \right), \end{array}$$ (24)

which is identical for the excitatory and inhibitory populations. The mean input to each neuron is given by [cf. Eq (3)],

$$\begin{array}{c} \mu =JK\left(1-\gamma g\right)\langle n\rangle , \end{array}$$ (25)

and, under the assumption of near-independence of the neurons, the variance of the inputs is well approximated by the sum of the variances from each sending neuron [cf. Eq (4)],

$$\begin{array}{c} \sigma^{2}=J^{2}K\left(1+\gamma g^{2}\right)\langle n\rangle \left(1-\langle n\rangle \right). \end{array}$$ (26)

Finally, the mean activity can be obtained from the self-consistency relation Eq (39).

Eq (22) shows that, when excitatory and inhibitory synaptic weights are scaled equally, the covariances scale with 1/*N* as long as the network feedback is strong (*W*_e_ ≫ 1), (for this argument, we assume that ⟨*n*⟩ is held constant, which may be achieved by adjusting a combination of *θ* and the external drive). Hence, conventional downscaling of population sizes tends to increase covariances.

**Fig 6:**
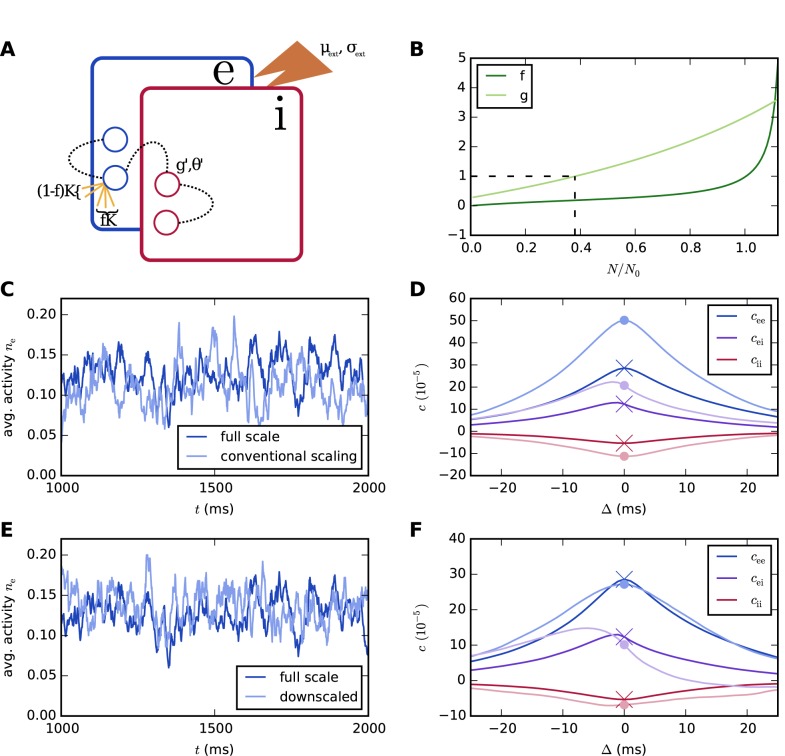
Binary network scaling that approximately preserves both mean activities and zero-lag covariances. **A** Increased covariances due to reduced network size can be countered by a change in the relative inhibitory synaptic weight combined with a redistribution of the synapses so that a fraction comes from outside the network. Adjusting a combination of the threshold and external drive restores the working point. **B** Scaling parameters versus relative network size for an example network. Since *γ* = 1 in this example, the scaling only works down to *g* = 1 (indicated by the horizontal and vertical dashed lines): Lower values of *g* only allow a silent or fully active network as steady states. **C**, **E** The mean activities are well preserved both by the conventional scaling in Eq (1) with an appropriate adjustment of *θ* (panel **C**), and by the method proposed here (panel **E**). **D**, **F** Conventional scaling increases the magnitude of zero-lag covariances in simulated data (panel **D**), while the proposed method preserves them (panel **F**). Dark colors: full-scale network. Light colors: downscaled network. Crosses and dots indicate zero-lag correlations in the full-scale and downscaled networks, respectively.

We use Eq (22) to perform a more sophisticated downscaling (cf. Fig 6). Let the new size of the excitatory population be *N*′. Eq (22) shows that the covariances can only be preserved when a combination of *W*_e_, *γ*, and *g* is adjusted. We take *γ* constant, and apply the transformation

$$\begin{array}{c} W_{e}\to fW_{e};\;g\to g^{\prime }. \end{array}$$ (27)

Solving Eq (22) for *f* and *g*′ yields (cf. Fig 6B)

$$\begin{array}{c} f=\frac{\frac{a\;c_{\text{ee}}}{N^{\prime }}+\frac{\gamma \;c_{\text{ii}}}{2}\left(c_{\text{ee}}-c_{\text{ii}}\right)}{W_{e}[(\frac{a}{N^{\prime }}+c_{\text{ee}})(\frac{a}{N}+\gamma c_{\text{ii}})-\frac{\gamma }{4}{\left(c_{\text{ee}}+c_{\text{ii}}\right)}^{2}]} \end{array}$$ (28)

$$\begin{array}{c} g^{\prime }=\frac{c_{\text{ee}}\left(c_{\text{ee}}-c_{\text{ii}}\right)-\frac{2a}{N^{\prime }}c_{\text{ii}}}{\gamma \;c_{\text{ii}}\left(c_{\text{ee}}-c_{\text{ii}}\right)+\frac{2a}{N^{\prime }}c_{\text{ee}}}. \end{array}$$ (29)

The change in *W*_e_ can be captured by *K* → *f* *K* as long as the working point (*μ*, *σ*) is maintained. This intuitively corresponds to a redistribution of the synapses so that a fraction *f* comes from inside the network, and 1 − *f* from outside (cf. Fig 6A). However, the external drive does not have the same mean and variance as the internal inputs, since it needs to make up for the change in *g*. The external input can be modeled as a Gaussian noise with parameters

$$\begin{array}{c} \mu_{\text{ext}}=KJ\left(1-\gamma g\right)\langle n\rangle -fKJ\left(1-\gamma g^{\prime }\right)\langle n\rangle \end{array}$$ (30)

$$\begin{array}{c} \sigma_{\text{ext}}^{2}=KJ^{2}\left(1+\gamma g^{2}\right)a-fKJ^{2}\left(1+\gamma g^{\prime 2}\right)a, \end{array}$$ (31)

independent for each neuron.

An alternative is to perform the downscaling in two steps: First change the relative inhibitory weights according to Eq (29) but keep the connection probability constant. The mean activity can be preserved by solving Eq (39) for *θ*, but the covariances are changed. The second step, which restores the original covariances, then amounts to redistributing the synapses so that a fraction $\tilde{f}$ comes from inside the network, and $1-\tilde{f}$ from outside, where the external (non-modeled) neurons have the same mean activity as those inside the network. This mean activity is negative, as the balanced regime implies stronger inhibition than excitation. Note that $\tilde{f}\neq f$, since *W*_e_ changes already in the first step.

The requirement that inhibition dominate excitation places a lower limit on the network size for which the scaling is effective. The reason is that *g* decreases with network size, so that a bifurcation occurs at *g* = 1/*γ*, beyond which the only steady states correspond to a silent network or a fully active one.

### Symmetric two-population spiking network

We have seen that the one-to-one relationship between effective connectivity and correlations does not hold in certain degenerate cases. Here we consider such a degenerate case and perform a scaling that preserves mean activities as well as both the size and the temporal structure of the correlations under reductions in both the number of neurons and the number of synapses. The network consists of one excitatory and one inhibitory population of LIF neurons with a population-independent connection probability and vanishing transmission delays. Due to the appearance of the eigenvalues in the numerator of the expression for the correlations in LIF networks [cf. Eqs (70) and (71)], such networks are subject to a reduced number of constraints when **W** has a zero eigenvalue, as this leaves a freedom to change the corresponding eigenvectors. Furthermore, identically vanishing delays greatly simplify the equations for the covariances.

The single-neuron and network parameters are as in Table 2 except that, here, *N* = 10,000, *J* = 0.2 mV, and the external drive is chosen such that the mean and standard deviation of the total input to each neuron are *μ* = 15 mV, *σ* = 10 mV. Furthermore, the delay is chosen equal to the simulation time step to approximate *d* = 0, which we assume here. The effective connectivity matrix for this network is

$$\begin{array}{c} W=w\;K\;(\begin{array}{cc} 1 & -\gamma g\\ 1 & -\gamma g \end{array}), \end{array}$$ (32)

where *w* = ∂*r*_target_/∂*r*_source_ is the effective excitatory synaptic weight obtained as the derivative of Eq (67). Here, we take into account the dependence of *w* on *J* to quadratic order. The inhibitory weight is approximated as *gw* to allow an analytical expression for the relative inhibitory weight in the scaled network to be derived. The left and right eigenvectors are ${\text{v}}^{1}=\frac{1}{\sqrt{1-\gamma g}}\;\left(\begin{array}{c} 1\\ -\gamma g \end{array}\right),$ ${\text{u}}^{1}=\frac{1}{\sqrt{1-\gamma g}}\;\left(\begin{array}{c} 1\\ 1 \end{array}\right)$ corresponding to eigenvalue *L* = *w* *K*(1 − *γ* *g*) and ${\text{v}}^{2}=\frac{1}{\sqrt{1-\frac{1}{\gamma g}}}\;\left(\begin{array}{c} 1\\ -1 \end{array}\right),$ ${\text{u}}^{2}=\frac{1}{\sqrt{1-\frac{1}{\gamma g}}}\;\left(\begin{array}{c} 1\\ \frac{1}{\gamma g} \end{array}\right)$ corresponding to eigenvalue 0. The normalization is chosen such that the bi-orthogonality condition Eq (47) is fulfilled.

A transformed connectivity matrix should have the same eigenvalues as **W**, and can thus be written as

$$\begin{array}{ccc} W^{\prime } & = & w^{\prime }K^{\prime }(\begin{array}{cc} 1 & -b\\ c & -bc \end{array}) \end{array}$$ (33)

$$\begin{array}{ccc} \text{where}\;b & = & \frac{1}{c}[1-\frac{wK}{w^{\prime }K^{\prime }}\left(1-\gamma g\right)]. \end{array}$$ (34)

Denote the new population sizes by *N*_1_ and *N*_2_. Equating the covariances before and after the transformation yields using Eq (71) and *A* ^*jk*^ = **v** ^*jT*^ **A** **v** ^*k*^ [cf. Eq (49)],

$$\begin{array}{ccc} & & \frac{\frac{a_{1}}{N}+\gamma g^{2}\frac{a_{2}}{N}}{{\left(1-\gamma g\right)}^{2}\left(2-2L\right)}(\begin{array}{cc} 1 & 1\\ 1 & 1 \end{array})+\frac{\frac{a_{1}}{N}+g\frac{a_{2}}{N}}{\left(2-\gamma g-\frac{1}{\gamma g}\right)\left(2-L\right)}(\begin{array}{cc} 1 & \frac{1}{\gamma g}\\ 1 & \frac{1}{\gamma g} \end{array})\\ & & \;=\frac{\frac{a_{1}}{N_{1}}+b^{2}\frac{a_{2}}{N_{2}}}{{\left(1-bc\right)}^{2}\left(2-2L\right)}(\begin{array}{cc} 1 & c\\ c & c^{2} \end{array})+\frac{\frac{a_{1}}{N_{1}}+\frac{b}{c}\frac{a_{2}}{N_{2}}}{\left(2-bc-\frac{1}{bc}\right)\left(2-L\right)}(\begin{array}{cc} 1 & \frac{1}{b}\\ c & \frac{c}{b} \end{array}). \end{array}$$ (35)

In Eq (35) we have assumed that the working points, and thus *a*_1_ and *a*_2_, are preserved, which may be achieved with an appropriate external drive as long as the corresponding variance remains positive. The four equations are simultaneously solved by

$$\begin{array}{ccc} N_{1} & = & \frac{Nw^{\prime }K^{\prime }a_{1}(2-L)}{wKga_{2}(wK-w^{\prime }K^{\prime })+a_{1}[2w^{\prime }K^{\prime }-wK\left(w^{\prime }K^{\prime }-\gamma gwK\right)]}\\ N_{2} & = & \frac{Na_{2}}{wK}\frac{L\left(L-w^{\prime }K^{\prime }-2\right)+2w^{\prime }K^{\prime }}{ga_{2}(2-L)+\left(w^{\prime }K^{\prime }-wK\right)\left(a_{1}+ga_{2}\right)}\\ c & = & 1, \end{array}$$ (36)

where *w*′ *K*′ may be chosen freely. Thus, the new connectivity matrix reads

$$\begin{array}{c} W^{\prime }=w^{\prime }\;K^{\prime }(\begin{array}{cc} 1 & \frac{wK}{w^{\prime }K^{\prime }}\left(1-\gamma \;g\right)-1\\ 1 & \frac{wK}{w^{\prime }K^{\prime }}\left(1-\gamma \;g\right)-1 \end{array}), \end{array}$$ (37)

which may also be cast into the form

$$\begin{array}{c} W^{\prime }=w^{\prime }\;K^{\prime }(\begin{array}{cc} 1 & -\gamma^{\prime }\;g^{\prime }\\ 1 & -\gamma^{\prime }\;g^{\prime } \end{array}), \end{array}$$ (38)

where *γ*′ = *N*_2_/*N*_1_ and $g^{\prime }=\frac{w^{\prime }K^{\prime }-L}{w^{\prime }K^{\prime }\gamma^{\prime }}$.

**Fig 7:**
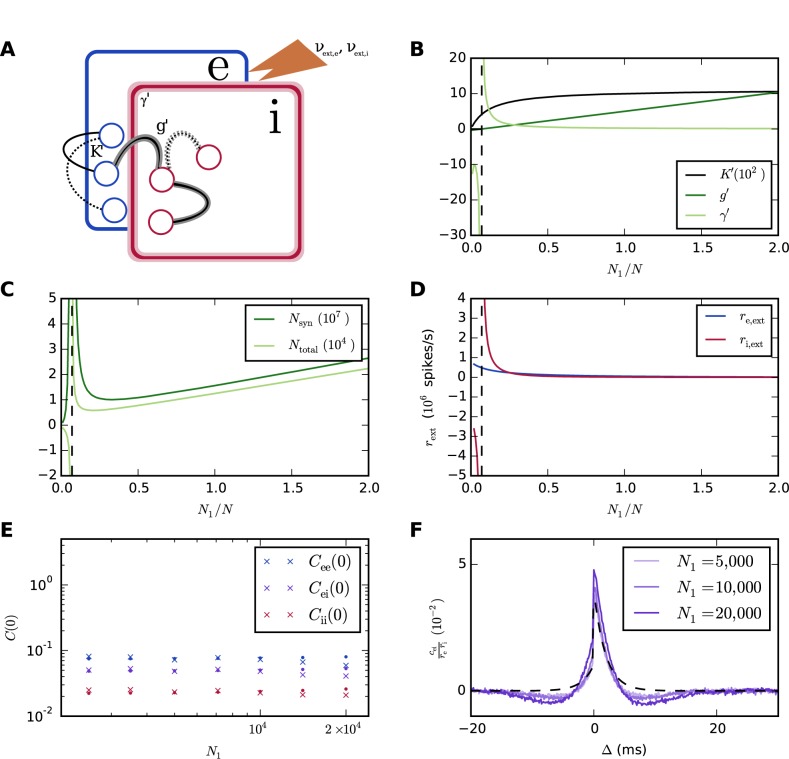
Spiking network scaling that approximately preserves mean firing rates and covariances. **A** Diagram illustrating the network and indicating the parameters that are adjusted. **B** Excitatory in-degrees *K*′, relative inhibitory synaptic weight *g*′, and relative number of inhibitory neurons *γ*′ versus scaling factor *N*_1_/*N*. The dashed vertical line indicates the limit below which the scaling fails. **C** Total number of neurons *N*_total_ = (1+*γ*′)*N*_1_ and total number of synapses *N*_syn_ = (1+*γ*′)^2^ *K*′ *N*_1_ versus scaling factor. **D** Rates of external excitatory and inhibitory Poisson inputs necessary for keeping firing rates constant. Average firing rates are between 23.1 and 23.5 spikes/s for both excitatory and inhibitory populations and all network sizes. **E** Integrated covariances, corresponding to zero-frequency components in the Fourier domain. Crosses: simulation results, dots: theoretical predictions. **F** Average covariance between excitatory-inhibitory neuron pairs for different network sizes. The dashed curve indicates the theoretical prediction for *N* = 10,000. Each network was simulated for 100 s.

When the populations receive statistically identical external inputs, we have *a*_1_ = *a*_2_ = *r*, since the internal inputs are also equal. Fig 7 illustrates the network scaling for the choice *w*′ = *w*. Results are shown as a function of the relative size *N*_1_/*N* of the excitatory population. External drive is provided at each network size to keep the mean and standard deviation of the total inputs to each neuron at the level indicated. The mean is supplied as a constant current input, while the variability is afforded by Poisson inputs according to Eqs (17) and (18) (Fig 7D). It is seen that the transformations (Fig 7B) are able to reduce both the total numbers of neurons and the total number of synapses (Fig 7C) while approximately preserving covariance sizes and shapes (Fig 7E,7F). Small fluctuations in the theoretical predictions in Fig 7E are due to the discreteness of numbers of neurons and synapses, and deviations of the effective inhibitory weight from the linear approximation *g* *w*. The fact that the theoretical prediction in Fig 7F misses the small dips around *t* = 0 may be due to the approximation of the autocorrelations by delta functions, eliminating the relative refractoriness due to the reset. The numbers of neurons and synapses increase again below some *N*_1_/*N*, and diverge as *g*′ becomes zero. This limits the scalability despite the additional freedom provided by the symmetry.

## Discussion

By applying and extending the theory of correlations in asynchronous networks of binary and networks of leaky integrate-and-fire (LIF) neurons, our present work shows that the scalability of numbers of neurons and synapses is fundamentally limited if mean activities and pairwise averaged activity correlations are to be preserved. We analytically derive a limit on the reducibility of the number of incoming synapses per neuron, *K* (the in-degree), which depends on the variance of the external drive, and which indirectly restricts the scalability of the number of neurons. Within these restrictive bounds, we propose a scaling of the synaptic strengths *J* and the external drive with *K* that can preserve mean activities and the size and temporal structure of pairwise averaged correlations. Mean activities can be approximately preserved by maintaining the mean and variance of the total input currents to the neurons, also referred to as the *working point*. The temporal structure of pairwise averaged correlations depends on the *effective connectivity*, a measure of the effective influence of source populations on target populations determined both by the physical connectivity and the working point of the target neurons. When the dependence of the effective connectivity on the synaptic strengths *J* is linearized, it can be written as *SJK*, where *S* is the susceptibility of the target neurons (quantifying the change in output activity for a unit change in input). Scalings and analytical predictions of pairwise averaged correlations are tested using direct simulations of randomly connected networks of excitatory and inhibitory neurons.

Our most important findings are:

1. The population-level effective connectivity matrix and pairwise averaged correlations are linked by a one-to-one mapping except in degenerate cases. Therefore, with few exceptions, any network scaling that preserves the correlations needs to preserve the effective connectivity.
2. The most straightforward way of simultaneously preserving mean activities and pairwise averaged correlations is to change the synaptic strengths in inverse proportion to the in-degrees (*J* ∝ 1/*K*), and to adjust the variance of the external drive to make up for the change in variance of inputs from within the network. Other scalings, such as $J\propto 1/\sqrt{K}$, can in principle also preserve both mean activities and pairwise averaged correlations, but then change the working point (hence the neuronal susceptibility determining the strength of stimulus responses, and the degree to which the activity is mean- or fluctuation-driven), and are analytically intractable for LIF networks due to the complicated dependence of the firing rates and the impulse response on the mean and variance of the inputs.
3. When downscaling the in-degrees *K* and scaling synaptic strengths as *J* ∝ 1/*K*, the variance of inputs from within the network increases, so that the variance of external inputs needs to be decreased to restore the working point. This is only possible up to the point where the variance of the external drive vanishes. The minimal in-degree scaling factor equals the ratio between the variance of inputs coming from within the network, and the total input variance due to both internal inputs and the external drive. The same limit to in-degree scaling holds more generally for scalings that simultaneously preserve mean activities and correlations. Thus, in the absence of a variable external drive, no downscaling is possible without changing mean activities, correlations, or both.
4. Within the identified restrictive bounds, the scaling *J* ∝ 1/*K*, where the external variance is adjusted to maintain the working point, can preserve mean activities and pairwise averaged correlations also in asynchronous networks deviating from the assumptions of the analytical theory presented here. We show this robustness for an example network with distributed in- and out-degrees and distributed synaptic weights, and for a network with non-Poissonian spiking.
5. For a sufficiently large change in in-degrees, a scaling that affects correlations can push the network from the linearly stable to an oscillatory regime or vice versa.
6. Transformations derived using the diffusion approximation are able to closely preserve the relevant quantities (mean activities, correlation shapes and sizes) in simulated networks of binary and spiking neurons within the given bounds. Reducing the number of neurons only increases correlation magnitudes without affecting their structure in this approximation.However, strong deviations from the assumptions of the diffusion approximation can cause also correlation structure to change in simulated networks under scalings originally constructed to maintain correlation structure. This occurs for instance when a drastic reduction in network size is coupled with a less than proportional reduction in in-degrees, leading to large numbers of common inputs and increased synchrony. Thus, the scalability of the number of neurons with available analytical results is indirectly limited by the minimal in-degree scaling factor.

In conclusion, we have identified limits to the reducibility of neural networks, even when only considering first- and second-order statistical properties. Networks are inevitably irreducible in some sense, in that downscaled networks are clearly not identical to their full-scale counterparts. However, mean activity, a first-order macroscopic quantity, can usually be preserved. The present work makes it clear that non-reducibility already sets in at the second-order macroscopic level of correlations. This does not imply a general minimal size for network models to be valid, merely that each network in question needs to be studied near its natural size to verify results from any scaled versions.

Our analytical theory is based on the diffusion approximation, in which inputs are treated as Gaussian noise, valid in the asynchronous irregular regime when activities are sufficiently high and synaptic weights are small. Moreover, external inputs are taken to be independent across populations, and delays and time constants are assumed to be unchanged under scaling. A further assumption of the theory is that the dynamics is stationary and linearly stable.

The one-to-one correspondence between effective connectivity and correlations applies with a few exceptions. For non-identical populations with different impulse responses, an analysis in the frequency domain demonstrates the equivalence under the assumption that the correlation matrix is invertible. An argument that assumes a diagonalizable effective connectivity matrix extends the equivalence to identical populations apart from cases where the effective connectivity matrix has eigenvalues that are zero or degenerate.

The equivalence of correlations and effective connectivity ties in with efforts to infer structure from activity, not only in neuroscience [59, 60, 61, 62, 63, 64, 65, 66] but also in other disciplines [67, 68, 69], as it implies that one should in principle be able to find the only—and therefore the real—effective connectivity that accounts for the correlations. Within the same framework as that used here, [65] shows that knowledge of the cross-spectrum at two distinct frequencies allows a unique reconstruction of the effective connectivity matrix by splitting the covariance matrix into symmetric and antisymmetric parts. The derivation considers a class of transfer functions (the Fourier transform of the neuronal impulse response) rather than any specific form, but the transfer function is taken to be unique, whereas the present work allows for differences between populations. Furthermore, we here present a more straightforward derivation of the equivalence, not focused on the practical aim of network reconstruction, and clarify the conditions under which reconstruction is possible.

In practice, using our results to infer structure from correlations may not be straightforward, due to both deviations from the assumptions of the theory and problems with measuring the relevant quantities. For instance, neural activity is often nonstationary [70], transfer functions are normally not measured directly, and correlations are imperfectly known due to measurement noise. Furthermore, inference of anatomical from functional connectivity (correlations) is often done based on functional magnetic resonance imaging (fMRI) measurements, which are sensitive only to very low frequencies and therefore only allow the symmetric part of the effective connectivity to be reliably determined [66]. The presence of unobserved populations providing correlated input to two or more observed populations can also hinder inference of network structure. Thus, high-resolution measurements (e.g., two-photon microscopy combined with optogenetics to record activity in a cell-type-specific manner [71, 72]) of networks with controlled input (e.g., in brain slices) hold the most promise for network reconstruction from correlations.

The effects on correlation-based synaptic plasticity of scaling-related changes in correlations may be partly compensated for by adjusting the learning parameters. For instance, an increase in average correlation size with factor 1/*N* without a change in temporal shape may be to some extent countered by reducing the learning rate by the same factor. Changes in the temporal structure of the correlations are more difficult to compensate for. When learning is linear or slow, so that the learning function can be approximated as constant (independent of the weights), the mean drift in the synaptic weights is determined by the integral of the product of the correlations and the learning function [73, 74]. Therefore, this mean drift may be kept constant under a change in correlation shapes by adjusting the learning function such that this product is preserved for all time lags. However, given that the expression for the correlations is a complicated function of the network parameters, the required adjustment of the learning function will also be complex. Moreover, the effects of this adjustment on precise patterns of weights are difficult to predict, since the distribution of correlations between neuron pairs may change under the proposed scalings, and this solution does not apply when learning is fast and weight-dependent.

The groundbreaking work of [46] identified a dynamic balance between excitation and inhibition as a mechanism for the asynchronous irregular activity in cortex, and showed that $J\propto 1/\sqrt{K}$ can robustly lead to a balanced state in the limit *N* → ∞ for constant *K*/*N*. However, it is not necessary to scale synaptic weights as $1/\sqrt{K}$ in order to obtain a balanced network state, even in the limit of infinite network size (and infinite *K*). For instance, *J* ∝ 1/*K* can retain balance in the infinite size limit in the sense that the sum of the excitatory and inhibitory inputs is small compared to each of these inputs separately. To retain irregular activity with this scaling one merely needs to ensure a variable external drive, as the internal variance vanishes for *N* → ∞. Moreover, in binary networks with neurons that have a Heaviside gain function (a hard threshold) identical across neurons, one does not even need a variable drive in order to stay in a balanced state [46, p. 1360]. This can be seen from a simple example of a network of *N* excitatory and *γN* inhibitory neurons with random connectivity with probability *p*, where *J* = *J*_0_/*N* > 0 is the synaptic amplitude of an excitatory synapse, and −*gJ* the amplitude of an inhibitory synapse. The network may receive a DC drive, which we absorb into the threshold *θ*. The summed input to each cell is then *μ* = *pNJ*(1 − *γg*) *n*, where *n* ∈ [0, 1] is the mean activity in the network. For a balanced state to arise, the negative feedback must be sufficiently strong, so that the mean activity *n* settles on a level where the summed input is close to the threshold *μ* ≃ *θ*. This will always be achieved if *pJ*_0_(1 − *γg*) < *θ* < 0: in a completely activated network (*n* = 1) the summed input is below threshold, and in a silent network (*n* = 0) the summed input is above threshold, hence the activity will settle close to the value *n* ≃ *θ*/[*pJ*_0_(1 − *γg*)]. As the variance of the synaptic input decreases with network size, the latter estimate of the mean activity will become exact in the limit *N* → ∞. The underlying reason for both 1/*K* and $1/\sqrt{K}$ scaling to lead to a qualitatively identical balanced state is the absence of a characteristic scale on which to measure the synaptic input: the threshold is hard. Only by introducing a characteristic scale, for example distributed values for the thresholds, the 1/*K* scaling with a DC drive will in the large *N* limit lead to a freezing of the balanced state due to the vanishing variance of the summed input, while with either $1/\sqrt{K}$ scaling, or 1/*K* scaling with a fluctuating external drive, the balanced state is conserved.

In [46], $J\propto 1/\sqrt{K}$ refers not only to a comparison between differently-sized networks, but also to the assumption that approximately $\sqrt{K}$ excitatory synapses need to be active to reach spike threshold. However, this is also not a necessary condition for balance, which can arise for a wide range of synaptic strengths relative to threshold, as long as inhibition is sufficiently strong compared to excitation. As discussed in “**Correlation-preserving scaling**”, with appropriately chosen external drive, *J* even drops out of the mean-field theory for binary networks with a Heaviside gain function altogether [52]. The difficulty in the interpretation of the [46] results illustrates a more general point: The primary goal of scaling studies is to identify the mechanisms governing network dynamics. Nevertheless, these studies usually also specify requirements on the robustness of the mechanism, leading to scaling laws for network parameters that may be more restrictive than a description of the mechanism per se. An example is the robustness to strong synapses, defined such that activation of $∼\sqrt{K}$ excitatory synapses suffices to reach threshold in the absence of an external drive [46, p. 1324]. This scenario was considered in order to create a condition under which dynamic balance is clearly *necessary* for achieving asynchronous irregular activity in balanced random networks, since combined inputs would otherwise far exceed the threshold. However, dynamic balance can arise also with weak synapses, e.g., with strength ∼ 1/*K* of the distance to threshold. Without questioning the value of scaling studies, which can distill essential mechanisms and are sometimes possible where finite-size analytical descriptions are intractable, this shows that scaling laws need to be interpreted with care.

The issue of the interrelation between network size, synaptic strengths, numbers of synapses per neuron, and activity is embedded in the wider context of anatomical and physiological scaling laws observed experimentally. In homeostatic synaptic plasticity, synaptic strengths are adjusted in a manner that keeps the activity of the postsynaptic neurons within a certain operating range [75, 76, 77]. Since postsynaptic activity depends not only on the strength of inputs but also on their number, this may induce a correlation between synaptic strengths and in-degree. In line with this hypothesis, excitatory postsynaptic currents (EPSCs) at single synapses were found to be inversely related to the density of active synapses onto cultured hippocampal neurons [78], and the size of both miniature EPSCs and evoked EPSCs between neurons decreased with network size and with the number of synapses per neuron in patterned cultures [79], although contrasting results have also been reported [80, 81]. In the development of a mammal, the neuronal network grows by orders of magnitude and is continuously modified. For instance, the amplitude of miniature EPSCs is reduced in a period of heightened synaptogenesis in rat primary visual cortex [82]. During such developmental processes, some functions are conserved and new functions emerge. This balance between stability and flexibility is an intriguing theoretical problem. Here, network scaling is deeply related to biological principles. Our results open up a new perspective for analyzing and interpreting such biological scaling laws.

Certainly, most network models will not fit neatly into the categories considered here, and detailed models often provide valuable insights regardless of whether they are scaled in a systematic manner. Nevertheless, it is usually possible to at least mention whether and how a particular model is scaled. When the results are not amenable to mathematical analysis, we suggest investigating through simulations of networks of different sizes how essential characteristics depend on numbers of neurons and synapses (the relevant characteristics depend on the model at hand, and do not necessarily include mean activities or correlations). Thus, while both the investigation of the infinity limit and the exploration of downscaled networks remain powerful methods of computational neuroscience, we argue for a more careful approach to network scaling than has hitherto been customary, making the type of scaling and its consequences explicit. Fortunately, in neuroscience full-scale simulations are now becoming routinely possible due to the technological advances of recent years.

## Methods

### Software

We verify analytical results for networks of binary neurons and networks of spiking neurons using direct simulations performed with NEST [83] revisions 10711 and 11264 for the spiking networks and revision 11540 for the binary networks. For simulating the multi-layer microcircuit model, PyNN version 0.7.6 (revision 1312) [84] was used with NEST 2.6.0 as back end, single-threaded on 12 MPI processes on a high-performance cluster. All simulations have a time step of 0.1 ms. Spike times in the microcircuit model are constrained to the grid. The other spiking network simulations use precise spike timing [85]. In part, Sage was used for symbolic linear algebra [86]. Pre- and post-processing and numerical analysis were performed with Python.

### Network structure and notation

For both the binary and the spiking networks, we derive analytical results where both the number of populations *N*_pop_ and the population-level connectivity are arbitrary. Specific examples are given of networks with a single, inhibitory population, or with two populations (one excitatory, one inhibitory) with either population-specific or population-independent connectivities. In addition, we discuss a multi-layer spiking cortical microcircuit model consisting of 77,169 neurons with approximately 3 × 10^8^ synapses, with eight populations (2/3E, 2/3I, 4E, 4I, 5E, 5I, 6E, 6I) and population-specific connection probabilities [58], slightly adjusted to enhance the asynchrony of the activity. The adjustments consist of replacing normally by lognormally distributed weights with the same mean and with coefficient of variation 3; and using 4.5 instead of 4 as the relative strength of synapses from 4I to 4E compared to excitatory synaptic strengths. Besides distributed synaptic strengths, the model has binomially distributed in- and out-degrees, and normally distributed delays (clipped at the simulation time step), thereby deviating from the assumptions of our analytic theory. It thus serves to evaluate the robustness of our analytical results to such deviations from the underlying assumptions.

In all cases, pairs of populations are randomly connected. In the binary and one- and two-population LIF network simulations, in-degrees are fixed and multiple directed connections between pairs of neurons (multapses) are disallowed. In the multi-layer microcircuit model, in-degrees are distributed and multapses are allowed. In case of population-specific connectivities, we denote the (unique or mean) in-degree for connections from population *β* to population *α* by *K*_*αβ*_, and synaptic strengths by *J*_*αβ*_. Population sizes are denoted by *N*_*α*_. For the example networks with population-independent connection probability, we denote the size of the excitatory population by *N*, the in-degree from excitatory neurons by *K* = *pN*, and the size of the inhibitory relative to the excitatory population by *γ*, so that the inhibitory in-degree is *γK*. Synaptic strengths are also taken to only depend on the source population, and are written as *J* for excitatory and −*gJ* for inhibitory synapses.

### Binary network dynamics

We denote the activity of neuron *j* by *n*_*j*_(*t*). The state *n*_*j*_(*t*) of a binary neuron is either 0 or 1, where 1 indicates activity, 0 inactivity [7, 42, 87]. The state of the network of *N* such neurons is described by a binary vector **n** = (*n*_1_, …, *n*_*N*_) ∈ {0,1}^*N*^. We denote the mean activity by ⟨*n*_*j*_(*t*)⟩_*t*_, where the average ⟨⟩_*t*_ is over time and realizations of the stochastic activity. The neuron model shows stochastic transitions (at random points in time) between the two states 0 and 1. In each infinitesimal interval [*t*, *t* + *δt*), each neuron in the network has the probability $\frac{1}{\tau }\delta t$ to be chosen for update [88], where *τ* is the time constant of the neuronal dynamics. We use an equivalent implementation in which the time points of update are drawn independently for all neurons. For a particular neuron, the sequence of update points has exponentially distributed intervals with mean duration *τ*, i.e., update times form a Poisson process with rate *τ* ^−1^. The stochastic update constitutes a source of noise in the system. Given that the *j*-th neuron is selected for update, the probability to end in the up state (*n*_*j*_ = 1) is determined by the gain function *F*_*j*_(**n**(*t*)) = Θ(∑_*k*_ *J*_*jk*_ *n*_*k*_(*t*) − *θ*) which in general depends on the activity **n** of all other neurons. Here *θ* denotes the threshold of the neuron and Θ(*x*) the Heaviside function. The probability of ending in the down state (*n*_*j*_ = 0) is 1 − *F*_*j*_(**n**). This model has been considered previously [42, 87, 89], and here we follow the notation introduced in [87] that we also employed in our earlier works. We skip details of the derivation here that are already contained in [9].

### First and second moments of activity in the binary network

The combined distribution of large numbers of independent inputs can be approximated as a Gaussian 𝓝(*μ*, *σ* ^2^) by the central limit theorem. The arguments *μ* and *σ* are the mean and standard deviation of the synaptic input noise, together referred to as the working point [cf. Eqs (3) and (4)]. The stationary mean activity of a given population of neurons then obeys [7, 9, 46, 52]

$$\begin{array}{ccc} \left\langle n\right\rangle & = & \left\langle F(n)\right\rangle \\ & ≃ & \int_{-\infty }^{\infty }\Theta \left(x-\theta \right)N\left(\mu ,\sigma^{2},x\right)dx\\ & = & \int_{\theta }^{\infty }N\left(\mu ,\sigma^{2},x\right)dx\\ & = & \frac{1}{2}\text{erfc}(\frac{\theta -\mu (\langle n\rangle )}{\sqrt{2}\sigma (\langle n\rangle )}). \end{array}$$ (39)

This equation needs to be solved self-consistently because ⟨*n*⟩ influences *μ*, *σ* through interactions within the population itself and with other populations.

When network activity is stationary, the covariance of the activities of a pair (*j*, *k*) of neurons is defined as *c*_*jk*_(Δ) = ⟨*δn*_*j*_(*t* + Δ)*δn*_*k*_(*t*)⟩_*t*_, where *δn*_*j*_(*t*) = *n*_*j*_(*t*) − ⟨*n*_*j*_(*t*)⟩_*t*_ is the deviation of neuron *j*’s activity from expectation, and Δ is a time lag. Instead of the raw correlation ⟨*n*_*j*_(*t* + Δ)*n*_*k*_(*t*)⟩_*t*_, here and for the spiking networks we measure the covariance, i.e., the second centralized moment, which is also identical to the second cumulant. To derive analytical expressions for the covariances in binary networks in the asynchronous regime, we follow the theory developed in [7, 9, 42, 52, 53]. We first consider the case of vanishing transmission delays *d* = 0 and then discuss networks with delays.

Let

$$\begin{array}{c} c_{\alpha \beta }=\frac{1}{N_{\alpha }N_{\beta }}\;\sum_{j\in \alpha ,k\in \beta ,j\neq k}c_{jk} \end{array}$$ (40)

be the covariance averaged over disjoint pairs of neurons in two (possibly identical) populations *α*, *β*, and $a_{\alpha }=\frac{1}{N_{\alpha }}\sum_{j\in \alpha }a_{j}$ the population-averaged single-neuron variance *a*_*j*_(Δ) = ⟨*δn*_*j*_(*t* + Δ)*δn*_*j*_(*t*)⟩_*t*_. Note that for *α* = *β* there are only *N*_*α*_(*N*_*α*_ − 1) disjoint pairs of neurons, so *c*_*αα*_ differs from the average pairwise cross-correlation by a factor (*N*_*α*_ − 1)/*N*_*α*_, but we choose this definition because it slightly simplifies the population-level equations. For sufficiently weak synapses and sufficiently high firing rates, and when higher-order correlations can be neglected, a linearized equation relating these quantities can be derived for the case *d* = 0 ([42] Eqs (9.14)–(9.16); [7] supplementary material Eq (36), [9] Eq (10)),

$$\begin{array}{c} 2c_{\alpha \beta }=\sum_{\gamma }(W_{\alpha \gamma }c_{\gamma \beta }+W_{\beta \gamma }c_{\gamma \alpha })+W_{\alpha \beta }\frac{a_{\beta }}{N_{\beta }}+W_{\beta \alpha }\frac{a_{\alpha }}{N_{\alpha }}. \end{array}$$ (41)

Here, we have assumed identical time constants across populations, and

$$\begin{array}{c} W_{\alpha \beta }=S\left(\mu_{\alpha },\sigma_{\alpha }\right)J_{\alpha \beta }K_{\alpha \beta } \end{array}$$ (42)

is the linearized effective connectivity. The susceptibility *S* is defined as the slope of the gain function averaged over the noisy input to each neuron [9, 52, 53], reducing for a Heaviside gain function to

$$\begin{array}{c} S\left(\mu ,\sigma \right)=\frac{1}{\sqrt{2\pi }\sigma }e^{-\frac{{\left(\mu -\theta \right)}^{2}}{2\sigma^{2}}}. \end{array}$$ (43)

With the definitions

$$\begin{array}{c} {\bar{c}}_{\alpha \beta }\equiv \frac{1}{N_{\alpha }N_{\beta }}\;\sum_{j\in \alpha ,k\in \beta }c_{jk}=c_{\alpha \beta }+\delta_{\alpha \beta }\frac{a_{\alpha }}{N_{\alpha }} \end{array}$$ (44)

$$\begin{array}{c} P_{\alpha \beta }\equiv \delta_{\alpha \beta }-W_{\alpha \beta } \end{array}$$ (45)

Eq (41) is recognized as a continuous Lyapunov equation

$$\begin{array}{c} P\bar{c}+{\left(P\bar{c}\right)}^{T}=2\text{diag}(\frac{a_{\alpha }}{N_{\alpha }})\equiv 2A, \end{array}$$ (46)

which can be solved using known methods. Let **v** ^*j*^,**u** ^*k*^ be the left and right eigenvectors of **W**, with eigenvalues *λ*_*j*_ and *λ*_*k*_, respectively. Choose the normalization such that the left and right eigenvectors are biorthogonal,

$$\begin{array}{c} v^{jT}u^{k}=\delta_{jk}. \end{array}$$ (47)

Then multiplying Eq (46) from the left with **v** ^*jT*^ and from the right with **v** ^*k*^ yields

$$\begin{array}{c} \left(1-\lambda_{j}\right)v^{jT}\bar{c}v^{k}+v^{jT}\bar{c}v^{k}\left(1-\lambda_{k}\right)=2v^{jT}Av^{k}. \end{array}$$ (48)

Define

$$\begin{array}{c} m^{jk}\equiv v^{jT}mv^{k}, \end{array}$$ (49)

for $\text{m}=\text{c},\bar{\text{c}},\text{A}$. Then solving Eq (48) for $\bar{\text{c}}$ gives

$$\begin{array}{c} \bar{c}=\sum_{j,k}\frac{2A^{jk}}{2-\lambda_{j}-\lambda_{k}}u^{j}u^{kT}, \end{array}$$ (50)

as can be verified using Eq (47). This provides an approximation of the population-averaged zero-lag correlations, including contributions from both auto- and cross-correlations.

To determine the temporal structure of the population-averaged cross-correlations, we start from the single-neuron level, for which the correlations approximately obey ([53] Eq (29))

$$\begin{array}{c} \tau \frac{d}{d\Delta }c_{jk}\left(\Delta \right)+c_{jk}\left(\Delta \right)=\sum_{i}w_{ji}c_{ik}\left(\Delta \right),\;\Delta \geq 0, \end{array}$$ (51)

where *w*_*ij*_ is the neuron-level effective connectivity (*w*_*ij*_ = *S*_*i*_ *J*_*ij*_ if a connection exists and *w*_*ij*_ = 0 otherwise). This equation also holds on the diagonal, *j* = *k*. To obtain the population-level equation, we use Eqs (40) and (44) and count the numbers of connections, which yields a factor *K*_*αβ*_ for each projection. Eq (51) then becomes

$$\begin{array}{c} \tau \frac{d}{d\Delta }\bar{c}\left(\Delta \right)=-P\bar{c}\left(\Delta \right),\;\Delta \geq 0. \end{array}$$ (52)

This step from the single-neuron to the population level constitutes an approximation when the out-degrees are distributed, but is exact for fixed out-degree [8, 53]. The correlations for Δ < 0 are determined by ${\bar{c}}_{\alpha \beta }(-\Delta )={\bar{c}}_{\beta \alpha }(\Delta )$. With the definition Eq (49), Eq (52) yields

$$\begin{array}{c} \tau \frac{d}{d\Delta }{\bar{c}}^{jk}\left(\Delta \right)=\left(\lambda_{j}-1\right){\bar{c}}^{jk}\left(\Delta \right)\;\Delta \geq 0. \end{array}$$ (53)

Using the initial condition for $\bar{\text{c}}$ from Eq (50) and multiplying Eq (53) by **u** ^*j*^ **u** ^*kT*^, summing over *j* and *k*, we obtain the solution

$$\begin{array}{c} \bar{c}\left(\Delta \geq 0\right)=\sum_{j,k}\frac{2A^{jk}}{2-\lambda_{j}-\lambda_{k}}u^{j}u^{kT}e^{\frac{\lambda_{j}-1}{\tau }\Delta }. \end{array}$$ (54)

The shape of the autocovariances is well approximated by that for isolated neurons, $\text{A}e^{-\frac{\Delta }{\tau }}$, with corrections due to interactions being *O*(1/*N*) [42]. Substituting this form in Eq (54) leads to

$$\begin{array}{c} c\left(\Delta \geq 0\right)=\sum_{j,k}\frac{2A^{jk}}{2-\lambda_{j}-\lambda_{k}}u^{j}u^{kT}e^{\frac{\lambda_{j}-1}{\tau }\Delta }-Ae^{-\frac{\Delta }{\tau }}, \end{array}$$ (55)

equivalent to [42] Eq (6.20). Note that this equation still needs to be solved self-consistently, because the variance of the inputs to the neurons, which goes into *S*(*μ*, *σ*), depends on the correlations. However, correlations tend to contribute only a small fraction of the input variance in the asynchronous regime (cf. [9] Fig 3D). The accuracy of the result Eq (55) is illustrated in Fig 3A for a network with parameters given in Table 1 by comparison with a direct simulation. Note that the delays were not zero but equal to the simulation time step of 0.1 ms, sufficiently small for the correlations to be well approximated by Eq (55).

Now consider arbitrary transmission delay *d* > 0, and let both *d* and the input statistics be population-independent. This case is most easily approached from the Fourier domain, where the population-averaged covariances including autocovariances can be approximated as [53]

$$\begin{array}{c} \bar{C}\left(\omega \right)={\left(H{\left(\omega \right)}^{-1}-W\right)}^{-1}2\tau A{\left(H{\left(-\omega \right)}^{-1}-W^{T}\right)}^{-1}. \end{array}$$ (56)

Here, *H*(*ω*) is the transfer function

$$\begin{array}{c} H\left(\omega \right)=\frac{e^{-i\omega d}}{1+i\omega \tau }, \end{array}$$ (57)

which is equal for all populations under the assumptions made. The transfer function is the Fourier transform of the impulse response, which is a jump followed by an exponential relaxation,

$$\begin{array}{c} h\left(t\right)=\Theta \left(t-d\right)\frac{1}{\tau }e^{-\frac{t-d}{\tau }}, \end{array}$$ (58)

where Θ is the Heaviside step function.

For the case of population-independent *H*(*ω*), Fourier back transformation to the time domain is feasible, and was performed in [53] for symmetric connectivity matrices. Here, we consider generic connectivity (insofar as consistent with equal *H*(*ω*)), and again use projection onto the eigenspaces of **W** to obtain a form similar to Eq (55), i.e., insert the identity matrix

$$\begin{array}{c} \sum_{j}u^{j}v^{jT}=𝟙 \end{array}$$ (59)

both on the left and on the right of Eq (56), and Fourier transform to obtain

$$\begin{array}{ccc} 2\pi \bar{c}\left(\Delta \right) & = & \int_{-\infty }^{+\infty }\bar{C}\left(\omega \right)e^{i\omega \Delta }d\omega \\ & = & \int_{-\infty }^{+\infty }e^{i\omega \Delta }\sum_{j,k}u^{j}\frac{1}{H{\left(\omega \right)}^{-1}-\lambda_{j}}2\tau v^{jT}Av^{k}\frac{1}{H{\left(-\omega \right)}^{-1}-\lambda_{k}}u^{kT}d\omega \\ & = & 2\tau \sum_{j,k}u^{j}u^{kT}\;A^{jk}\int_{-\infty }^{+\infty }f_{jk}\left(\omega \right)\;e^{i\omega \Delta }\;d\omega \\ \text{with}\;f_{jk}\left(\omega \right) & \equiv & \frac{1}{H{\left(\omega \right)}^{-1}-\lambda_{j}}\;\frac{1}{H{\left(-\omega \right)}^{-1}-\lambda_{k}}. \end{array}$$ (60)

In the third line of Eq (60), we used *A* ^*jk*^ = **v** ^*jT*^ **A** **v** ^*k*^ and collected the frequency-dependent terms for clarity. The exponential *e* ^*iω*Δ^ does not have any poles, so the only poles stem from *f*_*jk*_, which we denote by *z*_*l*_(*λ*_*j*_) and the corresponding residues by Res_*j*,*k*_[*z*_*l*_(*λ*_*j*_)]. We only need to consider Δ ≥ 0, since the solution for negative lags follows from $\bar{\text{c}}(\Delta )={\bar{\text{c}}}^{T}(-\Delta )$. The equation can then be solved by contour integration over the upper half of the complex plane, as the integrand vanishes at *ω* → +*i*∞. Stability requires that the poles of the first term of Eq (60) lie only in the upper half plane (note that the linear approximation we have employed only applies in the stable regime). The poles of the second term correspondingly lie in the lower half plane and hence need not be considered. For *d* > 0, the locations of the poles are given by [53] Eq (12),

$$\begin{array}{c} z_{l}\left(\lambda_{j}\right)=\frac{i}{\tau }-\frac{i}{d}W_{l}(\lambda_{j}\frac{d}{\tau }e^{d/\tau }), \end{array}$$ (61)

where *W*_*l*_ is the *l* ^th^ of the infinitely many branches of the Lambert-W function defined by *x* = *W*(*x*)*e* ^*W*(*x*)^ [90]. For *d* = 0, the poles are $z(\lambda_{j})=-\frac{i}{\tau }\left(\lambda_{j}-1\right)$. Using the residue theorem thus brings Eq (60) into the form

$$\begin{array}{ccc} \bar{c}\left(\Delta \geq 0\right) & = & 2\tau iI\left(\gamma \right)\sum_{j,k,l}u^{j}u^{kT}\;A^{jk}{\text{Res}}_{j,k}[z_{l}(\lambda_{j})]e^{iz_{l}(\lambda_{j})\Delta }\\ & = & \sum_{j,k,l}a_{jkl}u^{j}u^{kT}e^{iz_{l}(\lambda_{j})\Delta },\\ \text{with}\;a_{jkl} & \equiv & 2\tau iI\left(\gamma \right)A^{jk}{\text{Res}}_{j,k}[z_{l}(\lambda_{j})], \end{array}$$ (62)

where *I*(*γ*) = 1 is the winding number of the contour *γ* around the poles. To see that Eq (62) reduces to Eq (55) when *d* = 0, substitute the poles in the upper half plane $z(\lambda_{j})=-\frac{i}{\tau }\left(\lambda_{j}-1\right)$ with residues [*iτ*(2 − *λ*_*j*_ − *λ*_*k*_)]^−1^ and note that $\text{c}(\Delta )=\bar{\text{c}}(\Delta )-\text{A}(\Delta )$.

When the input statistics and hence transfer functions are population-specific, Eq (56) becomes

$$\begin{array}{ccc} \bar{C}\left(\omega \right) & = & {\left(𝟙-M(\omega )\right)}^{-1}D\left(\omega \right){\left(𝟙-M^{T}\left(-\omega \right)\right)}^{-1}, \end{array}$$ (63)

$$\begin{array}{ccc} D(\omega ) & \equiv & \text{diag}({\left\{\frac{2\tau_{\alpha }a_{\alpha }}{N_{\alpha }(1+\omega^{2}\tau_{\alpha }^{2})}\right\}}_{\alpha =1\ldots N_{\text{pop}}}), \end{array}$$ (64)

where *M*_*αβ*_(*ω*) = *H*_*αβ*_(*ω*)*W*_*αβ*_.

### Spiking network dynamics

The spiking networks consist of single-compartment leaky integrate-and-fire neurons with exponential current-based synapses. The subthreshold dynamics of neuron *i* is given by

$$\begin{array}{ccc} \tau_{m}\frac{dV_{i}}{dt} & = & -V_{i}+I_{i}\left(t\right),\\ \tau_{s}\frac{dI_{i}}{dt} & = & -I_{i}+\tau_{m}\sum_{j}J_{ij}s_{j}\left(t-d\right), \end{array}$$ (65)

where we have set the resting potential to zero without loss of generality, and absorbed the membrane resistance into the synaptic current *I*_*i*_, in line with previous works [45, 91]. Bringing back the corresponding parameters, the dynamics reads

$$\begin{array}{ccc} \tau_{m}\frac{d{\tilde{V}}_{i}}{dt} & = & -({\tilde{V}}_{i}-E_{L})+R_{m}{\tilde{I}}_{i}\left(t\right),\\ \tau_{s}\frac{d{\tilde{I}}_{i}}{dt} & = & -{\tilde{I}}_{i}+\tau_{s}\sum_{j}{\tilde{J}}_{ij}s_{j}\left(t-d\right). \end{array}$$ (66)

Thus, our scaled synaptic amplitudes *J*_*ij*_ in terms of the amplitudes ${\tilde{J}}_{ij}$ of the synaptic current due to a single spike are $J_{ij}=R_{\text{m}}\tau_{\text{s}}/\tau_{\text{m}}{\tilde{J}}_{ij}$. Here, *τ*_m_ and *τ*_s_ are membrane and synaptic time constants, *E*_L_ is the leak or resting potential, *R*_m_ is the membrane resistance, *d* is the transmission delay, ${\tilde{I}}_{i}=I_{i}/R_{\text{m}}$ is the total synaptic current, and $s_{j}=\sum_{k}\delta (t-t_{k}^{j})$ are the incoming spike trains. When *V*_*i*_ reaches a threshold *θ*, a spike is assumed, and the membrane potential is clamped to a level *V*_r_ for a refractory period *τ*_ref_. Threshold and reset potential in physical units are shifted by the leak potential ($\theta =\tilde{\theta }-E_{\text{L}}$, $V_{\text{r}}=\tilde{V_{\text{r}}}-E_{\text{L}}$), showing that the assumption *E*_L_ = 0 in Eq (65) does not limit generality. The intrinsic dynamics of the neurons in the different populations are taken to be identical, so that population differences are only expressed in the couplings.

### First and second moments of activity in the spiking network

An approximation of the stationary mean firing rate of LIF networks with exponential current-based synapses was derived in [91],

$$\begin{array}{ccc} r & = & {\left(\tau_{m}\sqrt{\pi }\int_{\frac{V_{r}-\mu }{\sigma }+\frac{\alpha }{2}\sqrt{\frac{\tau_{s}}{\tau_{m}}}}^{\frac{\theta -\mu }{\sigma }+\frac{\alpha }{2}\sqrt{\frac{\tau_{s}}{\tau_{m}}}}\Psi \left(s\right)\;ds\right)}^{-1},\\ \Psi (s) & = & e^{s^{2}}(1+\text{erf}(s)),\\ \alpha & = & \sqrt{2}|\zeta (\frac{1}{2})|, \end{array}$$ (67)

where the summed synaptic input is characterized by a Gaussian noise with first moment *μ* and second moment *σ* ^2^ based on the diffusion approximation, and *ζ* is the Riemann zeta function.

For the covariances, we follow and extend the theory developed in [45, 53], starting with the average influence of a single synapse. Assuming that the network is in the asynchronous state, and that synaptic amplitudes are small, the synaptic influences can be averaged around the mean activity *r*_*j*_ of each neuron *j*. These influences are characterized by linear response kernels *h*_*jk*_(*t*, *t*′) defined as the derivative of the density of spikes of spike train *s*_*j*_(*t*) of neuron *j* with respect to an incoming spike train *s*_*k*_(*t*′), averaged over realizations of the remaining incoming spike trains **s**\*s*_*k*_ that act as noise. In the stationary state, the kernel only depends on the time difference *t* − *t*′, giving

$$\begin{array}{c} {\left\langle s_{j}\left(t\right)|s_{k}\right\rangle }_{s\backslash s_{k}}=r_{j}+\int_{-\infty }^{t}h_{jk}\left(t-t^{\prime }\right)\left(s_{k}\left(t^{\prime }\right)-r_{k}\right)\;dt^{\prime }, \end{array}$$

$$\begin{array}{ccc} h_{jk}\left(t-t^{\prime }\right) & = & {\left\langle \frac{\delta s_{j}\left(t\right)}{\delta s_{k}\left(t^{\prime }\right)}\right\rangle }_{s\backslash s_{k}}\\ & \equiv & w_{jk}h\left(t-t^{\prime }\right), \end{array}$$ (68)

where *δs*_*j*_ ≡ *s*_*j*_ − *r*_*j*_ is the *j*-th centralized (zero-mean) spike train. Here, *w*_*jk*_ is the integral of *h*_*jk*_(*t* − *t*′), and *h*(*t* − *t*′) is a normalized function capturing its time dependence, which may be source- and target-specific. The dimensionless effective weights *w*_*jk*_ are determined nonlinearly by the synaptic strengths *J*_*jk*_, the single-neuron parameters, and the working point (*μ*_*j*_,*σ*_*j*_) (cf. [45] Eq (A.3) but note that *β* as given there has a spurious factor *J*). We approximate the impulse response by the form Eq (58), where *τ* is now an effective time constant depending on the working point (*μ*_*j*_,*σ*_*j*_) and the parameters of the target neurons. This form of the impulse response, corresponding to a low-pass filter, appears to be a good approximation in the noisy regime when the neuron fires irregularly. In the mean-driven regime (*μ* ≫ *σ*) the transfer function of the LIF neuron is known to exhibit resonant behavior with a peak close to its firing rate. In this regime a single exponential response kernel is expected to be a poor approximation (see, e.g., [92] Fig 1). In general, the source population dependence of Eq (58) comes in through the delay *d*, and the target population dependence through both *τ* and *d*.

As for binary networks with delays, the average pairwise covariance functions *c*_*ij*_(Δ) ≡ ⟨*δs*_*i*_(*t* + Δ)*δs*_*j*_(*t*)⟩_*t*_ are most conveniently derived starting from the frequency domain. In case of identical transfer functions for all populations, the matrix of average cross-covariances is given by [53] Eq (16) minus the autocovariance contribution,

$$\begin{array}{ccc} C(\omega ) & = & {\left(H{\left(\omega \right)}^{-1}-W\right)}^{-1}WAW^{T}{\left(H{\left(-\omega \right)}^{-1}-W^{T}\right)}^{-1}\\ & + & {\left(H{\left(\omega \right)}^{-1}-W\right)}^{-1}\text{WA}\\ & + & AW^{T}{\left(H{\left(-\omega \right)}^{-1}-W^{T}\right)}^{-1}. \end{array}$$ (69)

Here, **W** contains the effective weights of single synapses from population *β* to population *α* times the corresponding in-degrees, *w*_*αβ*_ *K*_*αβ*_; and **A** contains the population-averaged autocovariances, which we approximate as $\delta_{\alpha \beta }\frac{r_{\alpha }}{N_{\alpha }}$, with *r*_*α*_ the mean firing rate, as also done in [45]. In [53], Eq (69) was written using a more general diagonal matrix instead of **A**, to help clarify close similarities between binary and LIF networks and Ornstein-Uhlenbeck processes or linear rate models; however, for LIF networks, this diagonal matrix corresponds precisely to the autocovariance matrix. We chose the form Eq (69) because it separates terms that vanish at either *ω* → *i*∞ or *ω* → −*i*∞ depending on Δ. This facilitates Fourier back transformation, as contour integration with an appropriate contour can be used for each term.

To perform the Fourier back transformation, we apply the same method as used for the binary network. Let **v** ^*j*^,**u** ^*j*^ be the left and right eigenvectors of the connectivity matrix **W**, and *λ*_*j*_ the corresponding eigenvalues. Insert ∑_*j*_ **u** ^*j*^ **v** ^*jT*^ = 𝟙 into Eq (69) on the left and right, and Fourier transform,

$$\begin{array}{cc} 2\pi c(\Delta ) & =\int_{-\infty }^{+\infty }C\left(\omega \right)e^{i\omega \Delta }d\omega \\ & =\int_{-\infty }^{+\infty }e^{i\omega \Delta }\{\sum_{j,k}u^{j}\frac{\lambda_{j}}{H{\left(\omega \right)}^{-1}-\lambda_{j}}v^{jT}Av^{k}\frac{\lambda_{k}}{H{\left(-\omega \right)}^{-1}-\lambda_{k}}u^{kT}\\ & \;+\sum_{j,k}u^{j}\frac{\lambda_{j}}{H{\left(\omega \right)}^{-1}-\lambda_{j}}v^{jT}Av^{k}u^{kT}\\ & \;+\sum_{j,k}u^{j}v^{jT}Av^{k}\frac{\lambda_{k}}{H{\left(-\omega \right)}^{-1}-\lambda_{k}}u^{kT}\}d\omega . \end{array}$$ (70)

As for the binary case, we only need to consider Δ ≥ 0, as the solution for Δ < 0 is given by **c**(Δ) = **c** ^*T*^(−Δ). The contour can then be closed over the upper half plane, where the term containing only *H*(−*ω*) has no poles due to the stability condition. When Δ < *d*, the contour for the term containing only *H*(*ω*) can also be closed in the lower half plane where it has no poles, so that the corresponding integral vanishes. Analogously, the integral of the term with only *H*(−*ω*) vanishes when 0 > Δ > −*d*. Therefore, the second and third terms represent ‘echoes’ of spikes arriving after one transmission delay [53]. For Δ = 0 and *d* > 0, only the first term contributes, and the contour can be closed in either half plane. As before, the poles are given by Eq (61) for *d* > 0, and by $z(\lambda_{j})=\mp \frac{i}{\tau }\left(\lambda_{j}-1\right)$ for *d* = 0. The residue theorem yields a solution of the form Eq (62), the only difference being the precise form of the residues, and the fact that we here consider **c** as opposed to $\bar{\text{c}}$.

In the absence of delays, an explicit solution can again be derived. For Δ > 0, the poles inside the contour are $z(\lambda_{j})=-\frac{i}{\tau }(\lambda_{j}-1)$ corresponding to the terms with *H*(*ω*)^−1^. The residue corresponding to $\frac{\lambda_{j}}{H{\left(\omega \right)}^{-1}-\lambda_{j}}$ is $\frac{\lambda_{j}}{i\tau }$, and the term $\frac{\lambda_{k}}{H{\left(-\omega \right)}^{-1}-\lambda_{k}}$ is finite and evaluates at the pole to $\frac{\lambda_{k}}{2-\lambda_{j}-\lambda_{k}}$. Using *A* ^*jk*^ = **v** ^*jT*^ **A** **v** ^*k*^ we get

$$\begin{array}{c} c\left(\Delta >0\right)=\sum_{j,k}\frac{A^{jk}}{\tau }\frac{\lambda_{j}(2-\lambda_{j})}{2-\lambda_{j}-\lambda_{k}}u^{j}u^{kT}e^{\frac{\lambda_{j}-1}{\tau }\Delta }, \end{array}$$ (71)

which is reminiscent of but not identical to Eq (55) for the binary network. Note that Eq (71) for the LIF network corresponds to spike train covariances with the dimensionality of 1/*t* ^2^ due to [*A* ^*jk*^] = [1/*t*] and the factor 1/*τ*, whereas the covariances for the binary network are dimensionless.

The population-specific generalization of Eq (69) reads

$$\begin{array}{ccc} C(\omega ) & = & {\left(𝟙-M(\omega )\right)}^{-1}M\left(\omega \right)AM^{T}\left(-\omega \right){\left(𝟙-M^{T}\left(-\omega \right)\right)}^{-1}\\ & & +{\left(𝟙-M(\omega )\right)}^{-1}M\left(\omega \right)A\\ & & +AM^{T}\left(-\omega \right){\left(𝟙-M^{T}\left(-\omega \right)\right)}^{-1}, \end{array}$$ (72)

where **M**(*ω*) has elements *H*_*αβ*_(*ω*)*K*_*αβ*_ *w*_*αβ*_, as before. The covariance matrix including autocovariances can be more simply written as

$$\begin{array}{c} \bar{C}\left(\omega \right)={\left(𝟙-M(\omega )\right)}^{-1}A{\left(𝟙-M^{T}\left(-\omega \right)\right)}^{-1}. \end{array}$$ (73)

The only difference compared to the expression Eq (63) for the binary network is the form of the diagonal matrix, here analogous to white output noise in a linear rate model, whereas the binary network resembles a linear rate model with white noise on the input side, which is passed through the transfer function before affecting the correlations [53].

### Fluctuating rate equation and stability condition

An alternative description of the spiking dynamics can be obtained by considering a system of linear coupled rate equations that produces the same moments to second order as the spiking dynamics [53]. The convolution equation

$$\begin{array}{ccc} y_{j}\left(t\right) & = & \sum_{k}\int \;h_{jk}\left(t-t^{\prime }\right)y_{k}\left(t^{\prime }\right)\;dt^{\prime }+x_{j}\left(t\right)\\ \text{with}\; & & \\ \left\langle x_{j}\left(t\right)x_{k}\left(s\right)\right\rangle & = & \delta_{jk}\;r_{j}\;\delta \left(t-s\right), \end{array}$$ (74)

with pairwise uncorrelated white noises *x*_*j*_ and the response kernel *h*_*jk*_ given by Eq (68) can be shown to yield a cross-covariance matrix of the form Eq (69) by considering the Fourier transform of Eq (74), written in matrix notation as

$$\begin{array}{ccc} Y(\omega ) & = & H(\omega )\;W\;Y(\omega )+X(\omega ). \end{array}$$ (75)

We can expand the latter equation into eigenmodes by multiplying from the left with the left-sided eigenvector **v** ^*k*^ of **W** and by writing the general solution as a linear combination of right-sided eigenmodes **Y**(*ω*) = ∑_*j*_ *η*_*j*_(*ω*) **u** ^*j*^ to obtain (with the bi-orthogonality relation **v** ^*kT*^ **u** ^*j*^ = *δ*_*kj*_)

$$\begin{array}{ccc} \eta_{k}\left(\omega \right) & = & H\left(\omega \right)\;\lambda_{k}\eta_{k}\left(\omega \right)+v^{kT}X\left(\omega \right)\\ \eta_{k}\left(\omega \right) & = & \frac{1}{1-\lambda_{k}H\left(\omega \right)}\;v^{kT}X\left(\omega \right). \end{array}$$ (76)

The latter equation shows that the same poles *z*(*λ*_*k*_) that appear in the covariance function Eq (70) also determine the evolution of the effective rate equation. Moreover, transforming Eq (76) back to the time domain, we see with

$$\begin{array}{ccc} \eta_{k}\left(t\right) & = & i\sum_{\text{poles}\;z(\lambda_{k})}\text{Res}(\frac{1}{1-\lambda_{k}H\left(z\right)},\;z\left(\lambda_{k}\right))\;v^{k}X\left(z\right)\;e^{\;iz(\lambda_{k})\;t} \end{array}$$

that the eigenmodes have a time evolution determined by *e* ^*iz*(*λ*_*k*_)*t*^. Hence the imaginary part of the pole *z*(*λ*_*k*_) controls whether the mode is exponentially growing (Im(*z*) < 0) or decaying (Im(*z*) > 0), while the real part determines the oscillation frequency.

## Supporting Information

S1 Text Derivation of one-to-one relationship between effective connectivity and correlations for binary networks and networks consisting of populations with identical response properties. (PDF)
