# Supplementary material for: Scalability of Asynchronous Networks Is Limited by One-to-One Mapping between Effective Connectivity and Correlations
Source: PLoS Comput Biol. 2015 Sep 1;11(9):e1004490. doi: 10.1371/journal.pcbi.1004490 (PMC4556689; doi:10.1371/journal.pcbi.1004490)
Supplement: S1 Text — (PDF) [file pcbi.1004490.s001.pdf]

## Representation of eigenvalues and corresponding time scales in the correlations: $\mathbf{d} = \mathbf{0}$

We here show for networks with an identical transfer function across populations and without transmission delays that, apart from potential vanishing eigenvalues of the effective connectivity matrix $\mathbf{W}$ in LIF networks, all eigenvalues $\lambda_j$ are represented with their corresponding time scales in the covariances, a result we use in **"Correlations uniquely determine effective connectivity: population-independent transfer function, $\mathbf{d} = \mathbf{0}$"**.

The matrix of prefactors for the term with time dependence $\exp\left[(\lambda_j - 1)\Delta/\tau\right]$ in expressions (55) and (71) for the average pairwise covariances can be written as

$$\sum_k \mathbf{u}^j \frac{\mathbf{v}^{jT}\mathbf{D}\mathbf{v}^k}{2-\lambda_j-\lambda_k}\mathbf{u}^{kT},$$

where $\mathbf{D}$ is a diagonal matrix with

$$\mathbf{D} \quad = \quad \begin{cases} 2\mathbf{A} & \text{for binary} \\ \frac{\lambda_j(2-\lambda_j)}{\tau}\mathbf{A} & \text{for LIF} \end{cases}.$$

The $k$-dependence of $\lambda_k$ can be taken out of the sum by reintroducing the connectivity matrix and using $\mathbf{W}^T\mathbf{v}^k = \lambda_k \mathbf{v}^k$,

$$\sum_k \mathbf{u}^j \frac{\mathbf{v}^{jT}\mathbf{D}\mathbf{v}^k}{2-\lambda_j-\lambda_k}\mathbf{u}^{kT} = \mathbf{u}^j\mathbf{v}^{jT} \underbrace{\mathbf{D}\left[2-\lambda_j-\mathbf{W}^T\right]^{-1}}_{\equiv(\mathbf{B}^{-1})^T} \underbrace{\sum_k \mathbf{v}^k\mathbf{u}^{kT}}_{\mathbb{1}},$$

where we have also brought the other terms that do not depend on $k$ in front of the sum, and used the biorthogonality of the left and right eigenvectors $\mathbf{v}^T, \mathbf{u}$ of $\mathbf{W}$. For the time scale corresponding to $\lambda_j$ not to be represented, the above expression should vanish. We show as follows that this gives a contradiction, implying that all time scales must be represented. Since $\mathbf{u}^j$ is an eigenvector, it must have at least one nonzero entry, say for population $\alpha$. For the outer product $\left(\mathbf{u}^j \otimes \left[\mathbf{v}^{jT}\left(\mathbf{B}^{-1}\right)^T\right]\right)_{\alpha\beta} = \mathbf{u}^j_\alpha \left[\mathbf{v}^{jT}\left(\mathbf{B}^{-1}\right)^T\right]_\beta$ to vanish for all $\alpha, \beta$, the term $\left[\mathbf{v}^{jT}\left(\mathbf{B}^{-1}\right)^T\right]_\beta$ should thus vanish for all $\beta$. Both $\mathbf{B} = \mathbf{D}^{-1}\left(2-\lambda_j-\mathbf{W}\right)$ and $\mathbf{B}^{-1}$ are well-defined unless $\lambda_j = 0$ in a LIF network, or one or more populations are inactive, yielding vanishing entries in $\mathbf{D}$. Thus, the condition for the contribution to the covariance to vanish for all pairs of populations becomes $\mathbf{B}^{-1}\mathbf{v}^j = \mathbf{0}$ or $\mathbf{v}^j = \mathbf{B}\cdot\mathbf{0} = \mathbf{0}$, which is inconsistent with the fact that $\mathbf{v}^j$ is an eigenvector. Hence, time scales corresponding to all eigenvalues are represented in the covariances.

## Correlations uniquely determine effective connectivity: population-independent transfer function, $\mathbf{d} = \mathbf{0}$

In this section we show for both binary and LIF networks with population-independent input statistics and without delays that under fairly general conditions, the shapes of the average pairwise cross-covariances and their population structure uniquely determine the effective connectivity. This argument extends the one-dimensional example given in **"Correlations uniquely determine effective connectivity: a simple example"**. As before, we assume the transfer function $H(\omega)$ itself, and in particular the time constant $\tau$, to be unchanged under scaling. Furthermore, we exclude the trivial scenarios where one or more of the populations are inactive, or do not interact either with themselves or any other population. The covariance matrix $\mathbf{c}(\Delta, d = 0)$ is then given by (55) for binary networks and (71) for LIF networks. Since the dependence on the time interval $\Delta$ of each of these expressions is determined by the eigenvalues $\lambda_j$, any scaling transformation should keep these constant if it is to preserve the shape of the covariances. Even for a LIF network with $\lambda_j = 0$, where the corresponding term drops out of the sum, this eigenvalue needs to be preserved (the only exception being that it may become equal to another existing eigenvalue), since otherwise an additional time dependence would appear. Besides $\exp[(\lambda_j - 1)\Delta/\tau]$, the prefactor of this term should be unchanged for each $j$ at least if there are no degenerate or vanishing eigenvalues, as each exponential function contributes a fall-off with a unique characteristic time scale to the sums in (55) and (71). For populations $\alpha, \beta$, these prefactors can be written as $\sum_k a_{jk} u^j_\alpha u^{kT}_\beta$ for both binary and LIF networks, where $a_{jk}$ is a scalar that depends on $\lambda_j$ and $\lambda_k$. To preserve the population structure of the covariances under any

scaling transformation, also the ratio $\sum_k a_{jk} u^j_{\alpha_1} u^{kT}_\beta / \sum_k a_{jk} u^j_{\alpha_2} u^{kT}_\beta$ should be unchanged. As shown in **"Representation of eigenvalues and corresponding time scales in the correlations: $\mathbf{d} = \mathbf{0}$"**, with the exception of LIF networks with $\lambda_j = 0$, there is always at least one pair of populations $\alpha_2, \beta$ with interactions on the time scale corresponding to $\lambda_j$, such that this ratio is well-defined and equals $u^j_{\alpha_1}/u^j_{\alpha_2}$. That is, the eigenvector entries should be preserved relative to each other, fixing the eigenvectors up to a scaling factor. Assuming that $\mathbf{W}$ is diagonalizable, the combined conditions on the eigenvalues and eigenvectors fix the effective connectivity matrix via $\mathbf{W} = \mathbf{U}\text{diag}(\lambda_1, \ldots, \lambda_{N_\text{pop}})\mathbf{U}^{-1}$ where $\mathbf{U} = \left(\mathbf{u}^1, \ldots, \mathbf{u}^{N_\text{pop}}\right)$ is the matrix of right eigenvectors of $\mathbf{W}$.

Thus, correlation structure uniquely determines the effective connectivity matrix at least if it is diagonalizable, and if its eigenvalues are neither zero nor degenerate.

## Correlations uniquely determine effective connectivity: population-independent transfer function, general d

Here we extend the argument of the previous sections to networks with transmission delays. To this end, it is again convenient to work in the Fourier domain. Since the Fourier transform is an isomorphism, the conclusions hold also in the time domain. The rate equation (75) for the LIF dynamics can be solved for the rates $\mathbf{Y}(\omega)$ as

$$\mathbf{Y}(\omega) = \frac{1}{1 - H(\omega)\,\mathbf{W}} \mathbf{X}(\omega),$$

while for binary networks we obtain [53]

$$\mathbf{Y}(\omega) = \frac{H(\omega)}{1 - H(\omega)\,\mathbf{W}} \mathbf{X}(\omega),$$

where $\mathbf{X}(\omega)$ is Gaussian white noise with amplitude determined by the autocorrelations, and the transfer function $H(\omega)$ is given by (57). For both types of networks, the dynamics can be decomposed into eigenmodes

$$\mathbf{Y}(\omega) = \sum_k \eta_k(\omega)\,\mathbf{u}^k,$$

with $\mathbf{u}^k$ the right-sided eigenvectors of $\mathbf{W}$. Let

$$\gamma(\omega) \quad = \quad \begin{cases} H(\omega) & \text{for binary} \\ 1 & \text{for LIF} \end{cases}.$$

In terms of the left-sided eigenvectors $\mathbf{v}^k$ of $\mathbf{W}$, the coefficients $\eta_k(\omega)$ are then given by (cf. (76))

$$\eta_k(\omega) = \mathbf{v}^{kT}\,\mathbf{Y}(\omega) \quad = \quad \frac{\gamma(\omega)}{1 - H(\omega)\lambda_k}\,\mathbf{v}^{kT}\,\mathbf{X}(\omega).$$

Assuming that $\mathbf{W}$ has no degenerate eigenvalues and that $\mathbf{X}(\omega)$ is nonzero for all populations (no inactive populations), the power spectrum of each component, $\langle \eta_k(-\omega)\eta_k(\omega)\rangle \propto \left|\frac{\gamma(\omega)}{1-H(\omega)\lambda_k}\right|^2$ has a unique shape.

As before, $\lambda_k = 0$ in a LIF network presents a special case: The spectrum of its coefficient reduces to a constant in the Fourier domain, corresponding to a delta function in the time domain. This mode only contributes to the autocovariances and not the cross-covariances, leaving the freedom to change the corresponding right-sided eigenvector without affecting the cross-covariances, consistent with the example in **"Symmetric two-population spiking network"**. However, such transformations also preserve the autocovariances, despite the change in the population structure of the contribution from the $\lambda_k = 0$ mode. This becomes clear by rewriting the rate equation for the LIF network as

$$\mathbf{Y}(\omega) = \mathbf{X}(\omega) + \frac{H(\omega)\,\mathbf{W}}{1 - H(\omega)\,\mathbf{W}} \mathbf{X}(\omega),$$

showing that the part of $\bar{\mathbf{C}}(\omega)$ corresponding to the delta peak in the time domain remains

$$\bar{\mathbf{C}}_\delta = \langle \mathbf{X}(\omega)\mathbf{X}^T(-\omega)\rangle,$$

as the fact that $\mathbf{X}(\omega)$ is white noise ensures that the expression above is just a constant, independent of $\omega$. Hence, symmetric LIF networks where one of the eigenvalues of $\mathbf{W}$ is zero form an exception to the rule that the correlations uniquely determine the effective connectivity.

Apart from this exception, the argument continues as follows. The power spectra together make up the covariance matrix in the Fourier domain

$$\begin{aligned}\bar{\mathbf{C}}(\omega) &= \langle \mathbf{Y}(-\omega)\mathbf{Y}(\omega)^T\rangle \\ &= \sum_{j,k}\langle \eta_j(-\omega)\eta_k(\omega)\rangle \mathbf{u}^j\mathbf{u}^{kT}.\end{aligned}$$

If $\mathbf{W}$ is diagonalizable, the $\mathbf{u}^k$ are linearly independent. Therefore, $\mathbf{u}^k\mathbf{u}^{kT}$ cannot be expressed as a linear combination of the remaining terms $\mathbf{u}^j\mathbf{u}^{kT}$. It thus suffices to consider the contribution of a single mode

$$\langle \eta_k(-\omega)\eta_k(\omega)\rangle \mathbf{u}^k\mathbf{u}^{kT},$$

as its population structure makes a unique contribution to the covariance matrix. If the covariance matrix is to be preserved, the latter term must hence be preserved. This implies that $\lambda_k$ cannot change, since it governs the covariance shape as a function of $\omega$, and hence the temporal structure. Since $\mathbf{u}^k$ is by definition an eigenvector, it has at least one non-vanishing component, say $u^k_\alpha \neq 0$. Then the $\alpha$-th row of the outer product,

$$u^k_\alpha\,(u^k_1,\ldots,u^k_{N_\mathrm{pop}}),$$

must be preserved (except for $\lambda_k = 0$ in a LIF network, as explained above). At the $\alpha$-th column the entry is $\left(u^k_\alpha\right)^2$, so $u^k_\alpha$ can only differ by a factor $\rho \in \{-1,+1\}$. The conservation of the remaining entries $u^k_\alpha u^k_\beta$, $\beta \neq \alpha$ implies that the $u^k_\beta$ are multiplied by the same factor $\rho$. Hence the eigenvector must have the same direction. As before, by the diagonalizability of $\mathbf{W}$, the temporal and population structure of the correlations thus fix the effective connectivity matrix via $\mathbf{W} = \mathbf{U}\mathrm{diag}(\lambda_1,\ldots,\lambda_{N_\mathrm{pop}})\mathbf{U}^{-1}$ with $\mathbf{U} = \left(\mathbf{u}^1,\ldots,\mathbf{u}^{N_\mathrm{pop}}\right)$ the matrix of right eigenvectors of $\mathbf{W}$.

## Correlations uniquely determine effective connectivity: population-dependent transfer function, binary networks

In **"Correlations uniquely determine effective connectivity: the general case"**, we demonstrated a one-to-one correspondence between the effective connectivity and the correlations for LIF networks with non-identical populations. We here show that the same result is obtained for binary networks using analogous arguments. As before, we assume the summed cross- and auto-covariance matrix in frequency domain $\bar{\mathbf{C}}(\omega) = \mathbf{C}(\omega) + \mathbf{A}(\omega)$ to be invertible, and we expand the inverse of (63) with $\mathbf{D}(\omega) = \mathbf{H}(\omega)\mathbf{D}\mathbf{H}(-\omega)$ and $\mathbf{H}(\omega) = \mathrm{diag}\left(\{H_\alpha(\omega)\}_{\alpha=1\ldots N_\mathrm{pop}}\right)$ to obtain the diagonal element

$$\begin{aligned}\bar{C}^{-1}_{\alpha\alpha} &= \frac{1+\omega^2\tau_\alpha^2}{D_\alpha} \\ &- \frac{W_{\alpha\alpha}}{D_\alpha}\left(e^{-i\omega d_{\alpha\alpha}}(1-i\omega\tau_\alpha) + e^{i\omega d_{\alpha\alpha}}(1+i\omega\tau_\alpha)\right) \\ &+ \sum_\gamma \frac{W^2_{\gamma\alpha}}{D_\gamma}.\end{aligned}$$

Since $D^{-1}_\alpha$ determines a quadratic dependence on $\omega$ that cannot be offset by other terms, it needs to be preserved. This fixes $W_{\alpha\alpha}$, which similarly determines a unique $\omega$-dependence. Furthermore, we have for $\alpha \neq \beta$

$$
\begin{aligned}
\bar{C}^{-1}_{\alpha\beta} &= \frac{W_{\alpha\beta}}{D_\alpha} e^{-i\omega d_{\alpha\beta}} \left(-1 + i\omega\tau_\alpha + W_{\alpha\alpha} e^{i\omega d_{\alpha\alpha}}\right) \\
&+ \frac{W_{\beta\alpha}}{D_\beta} e^{i\omega d_{\beta\alpha}} \left(-1 - i\omega\tau_\beta + W_{\beta\beta} e^{-i\omega d_{\beta\beta}}\right) \\
&+ \sum_{\gamma \neq \alpha,\beta} \frac{W_{\gamma\alpha} W_{\gamma\beta}}{D_\gamma} e^{i\omega(d_{\gamma\alpha} - d_{\gamma\beta})}.
\end{aligned}
$$

Here, the term $W_{\alpha\beta} D_\alpha^{-1} e^{-i\omega d_{\alpha\beta}} i\omega\tau_\alpha$ cannot be offset by other terms unless $d_{\alpha\beta} = d_{\beta\alpha} = 0$, showing that $W_{\alpha\beta}$ needs to be unchanged in order to keep $\bar{C}^{-1}_{\alpha\beta}$ constant. In contrast to the LIF case, $\mathbf{C}(\omega)$ differs from $\bar{\mathbf{C}}(\omega)$ not by constant terms, but by $\mathrm{diag}\left(\left\{\frac{2\tau_\alpha}{1+\omega^2\tau_\alpha^2} \frac{a_\alpha}{N_\alpha}\right\}_{\alpha=1\ldots N_{\mathrm{pop}}}\right)$. Therefore, a priori it appears that there may be a freedom to scale both the population sizes $N_\alpha$ and terms in $\bar{\mathbf{C}}(\omega)$ with the same inverse quadratic $\omega$-dependence. We can see what this entails by considering

$$
\begin{aligned}
\bar{\mathbf{Q}}(\omega) &\equiv \bar{\mathbf{C}}(\omega) \mathrm{diag}\left(\left\{1 + \omega^2\tau_\alpha^2\right\}_{\alpha=1\ldots N_{\mathrm{pop}}}\right) \\
&= \mathbf{C}(\omega) \mathrm{diag}\left(\left\{1 + \omega^2\tau_\alpha^2\right\}_{\alpha=1\ldots N_{\mathrm{pop}}}\right) \\
&+ \mathrm{diag}\left(\left\{\frac{2\tau_\alpha a_\alpha}{N_\alpha}\right\}_{\alpha=1\ldots N_{\mathrm{pop}}}\right).
\end{aligned}
$$

This shows that changing any $\omega$-dependent terms in $\bar{\mathbf{Q}}(\omega)$ would change the $\omega$-dependence of $\mathbf{C}(\omega)$. Furthermore, the elements of $\bar{\mathbf{Q}}^{-1}(\omega)$ have the same form as those of $\bar{\mathbf{C}}^{-1}(\omega)$ for the LIF network except for the index of $\tau_\alpha$, with diagonal elements

$$
\begin{aligned}
\bar{Q}^{-1}_{\alpha\alpha}(\omega) = & \frac{1}{D_\alpha} \\
& - \frac{W_{\alpha\alpha}}{D_\alpha} \left(\frac{e^{-i\omega d_{\alpha\alpha}}}{1 + i\omega\tau_\alpha} + \frac{e^{i\omega d_{\alpha\alpha}}}{1 - i\omega\tau_\alpha}\right) \\
& + \sum_\gamma \frac{1}{D_\gamma} \frac{W_{\gamma\alpha}^2}{1 + \omega^2\tau_\alpha^2},
\end{aligned}
$$

and off-diagonal elements

$$
\begin{aligned}
\bar{Q}^{-1}_{\alpha\beta} &= \frac{W_{\alpha\beta}}{D_\alpha} e^{-i\omega d_{\alpha\beta}} \left(-\frac{1}{1 + i\omega\tau_\alpha} + W_{\alpha\alpha} \frac{e^{i\omega d_{\alpha\alpha}}}{1 + \omega^2\tau_\alpha^2}\right) \\
&+ \frac{W_{\beta\alpha}}{D_\beta} e^{i\omega d_{\beta\alpha}} \left(-\frac{1}{1 + \omega^2\tau_\alpha^2}(1 + i\omega\tau_\beta) + W_{\beta\beta} \frac{e^{-i\omega d_{\beta\beta}}}{1 + \omega^2\tau_\alpha^2}\right) \\
&+ \sum_{\gamma \neq \alpha,\beta} \frac{W_{\gamma\alpha} W_{\gamma\beta}}{D_\gamma} \frac{e^{i\omega(d_{\gamma\alpha} - d_{\gamma\beta})}}{1 + \omega^2\tau_\alpha^2}.
\end{aligned}
$$

Hence, comparing to (14), we reach the same conclusion as for the LIF network: in order to preserve $\mathbf{C}(\omega)$, $\mathbf{D}$ and $\mathbf{W}$ must not change, at least if all connections exist, and if there are no symmetries in the delays and time constants like those described in **"Correlations uniquely determine effective connectivity: the general case"**.
